# Scalable photonic reservoir computing for parallel machine learning tasks

**DOI:** 10.1038/s41467-025-67983-z

**Published:** 2025-12-31

**Authors:** A. Aadhi, L. Di Lauro, B. Fischer, P. Dmitriev, I. Alamgir, C. Mazoukh, N. Perron, E. A. Viktorov, A. V. Kovalev, A. Eshaghi, S. Vakili, M. Chemnitz, P. Roztocki, B. E. Little, S. T. Chu, D. J. Moss, R. Morandotti

**Affiliations:** 1 Institut National de la Recherche Scientifique - Énergie Matériaux Télécommunications, Varennes, QC Canada; 2 Leibniz Institute of Photonic Technology, Jena, Germany; 3 ITMO University, St Petersburg, Russia; 4 Huawei Technologies Canada, Markham, ON Canada; 5 QXP Technology Inc, Xi’an, China; 6 City University of Hong Kong, Hong Kong, China; 7 Optical Sciences Centre, Swinburne University of Technology, Hawthorn, VIC Australia

**Keywords:** Applied optics, Nonlinear optics, Photonic devices, Fibre optics and optical communications

## Abstract

Neuromorphic photonics enables brain-inspired information processing with higher bandwidth and lower energy consumption than traditional electronics, addressing the growing computational demands of the Internet of Things, cloud services, and edge computing. However, even current state-of-the-art electronic and photonic platforms are incapable of delivering the scalable throughput, multitasking processing, and energy efficiency required by these applications. Here, we demonstrate a tunable photonic reservoir computing device based on a nonlinear amplifying loop mirror (NALM), leveraging a time-delayed, single-unit, all-optical architecture. By combining dense temporal encoding with wavelength-division multiplexing, the system supports concurrent multitasking across independent data channels, enabling scalable computational performance without additional hardware complexity. Experiments and theoretical validation on classification and prediction benchmarks demonstrate the device’s performance, achieving a throughput of 20 tera-operations-per-second and an energy efficiency of 4.4 fJ per operation. These results highlight a promising path towards reconfigurable, compact, and high-performance photonic processors for real-time intelligent applications.

## Abstract (web summary)

Neuromorphic computing processes data faster and with less energy than electronics. Here, authors demonstrate a reconfigurable photonic reservoir computer that performs multiple machine learning tasks in parallel at ultrafast rates while using extremely low energy per operation.

## Introduction

The rapid evolution of artificial intelligence (AI) and machine learning (ML) has revolutionized data processing, driving demand for computational platforms that are capable of handling vast amounts of data with high processing speed and minimal latency^1,2^. Neuromorphic photonics presents a promising solution to these challenges^2–8^ as it leverages optical systems to emulate neural networks, thus providing higher bandwidth, faster speeds, lower energy consumption, and smaller footprints compared to traditional electronic processors^7,9–11^. This solution can also lower carbon emissions, especially when training on large datasets. However, existing photonic architectures have critical limitations in computational throughput, energy efficiency, and latency, thus hindering their ability to meet the growing computational needs of AI, deep learning^12^, and other real-time-based applications^13,14^. Neuromorphic computing frameworks, such as extreme learning machines^15^, wave-based neural networks^16^, and reservoir computing (RC)^17,18^, have emerged as compelling approaches for the efficient implementation of photonic processing devices for ML.

Optical RC systems exploit physical dynamics to process input data, thus reducing training time and complexity while improving computational efficiency. To date, single-node optical RC architectures have been demonstrated using active components (such as semiconductor lasers^19^, amplifiers^20,21^, and electro-optic modulators^22,23^), as well as passive components^24^ (including saturable absorbers^25,26^ and photodiodes^27^). Despite significant progress, current RC implementations remain limited by their inherent architecture, in which nodes are created and interconnected through feedback mechanisms^27,28^. These mechanisms simplify the design by generating virtual nodes, but also introduce latency and limit processing speed, which is defined as system throughput (i.e., the total number of operations per second across all input data streams within a single reservoir unit). Recent efforts to increase computational throughput in RC have focused on faster electro-optical modulation and advanced data encoding schemes, such as pulse-amplitude modulation^29–31^ (PAM), often paired with wavelength-division multiplexing (WDM). However, these methods frequently rely on sequential sweeping across WDM channels^32–34^, or time-interleaving techniques^35–39^, which both introduce throughput latency and prevent the concurrent execution of independent tasks. Additional attempts at further scaling up performance have involved adding components or increasing the number and dimensionality of computational layers and readout components^5,39^. Typically, these result in decreased energy efficiency and processing speeds. Furthermore, existing WDM-based RC architectures^21,38,39^ also have limited tunability, when design constraints inherently fix both the reservoir memory and the activation function. At the same time, nonlinearity is introduced electronically at the input/output stage, resulting in optical-to-electrical-to-optical (OEO) latency and thus limiting the richness of reservoir dynamics. Such constraints significantly reduce system flexibility and adaptability for diverse ML tasks. Additionally, architectures employing spectral mixing approaches^21^ encounter nonlinear crosstalk^40^, which, in turn, limits the number of independently encoded channels and consequently degrades system accuracy and scalability.

To address these challenges, we demonstrate an RC device based on a nonlinear amplifying loop mirror (NALM) architecture. We leverage the NALM configuration for its proven ability to generate highly stable and nonlinear dynamics^41–44^, which are essential for optical reservoir computing. These dynamics enhance the reservoir’s computational capacity by enabling complex input mapping into a high-dimensional state space, thereby improving sensitivity to extremely small signal variations^45,46^. The intrinsic stability of the NALM ensures consistent classification performance, even under noisy or fluctuating input conditions.

Our device features an integrated nonlinear spiral waveguide (SW) and a semiconductor optical amplifier (SOA), both of which modulate the NALM overall nonlinear transfer function (i.e., the reservoir activation function). In contrast to previous RC implementations that rely on SOAs operating in saturation, whether in unidirectional configurations^47–49^ or through cross-gain modulation^50,51^, we set the SOA below saturation for all our experiments. Saturated SOAs exhibit plateaued gain, limiting the dynamic range and tunability of the nonlinear response. The unsaturated regime, on the other hand, allows for precise tunability of the gain and, therefore, of the reservoir activation function. Furthermore, by adjusting the intensity of the propagating field using a variable optical attenuator (VOA), we achieve additional control over the reservoir’s fading memory response, significantly enhancing both versatility and stability for diverse computational tasks.

**Fig. 1:**
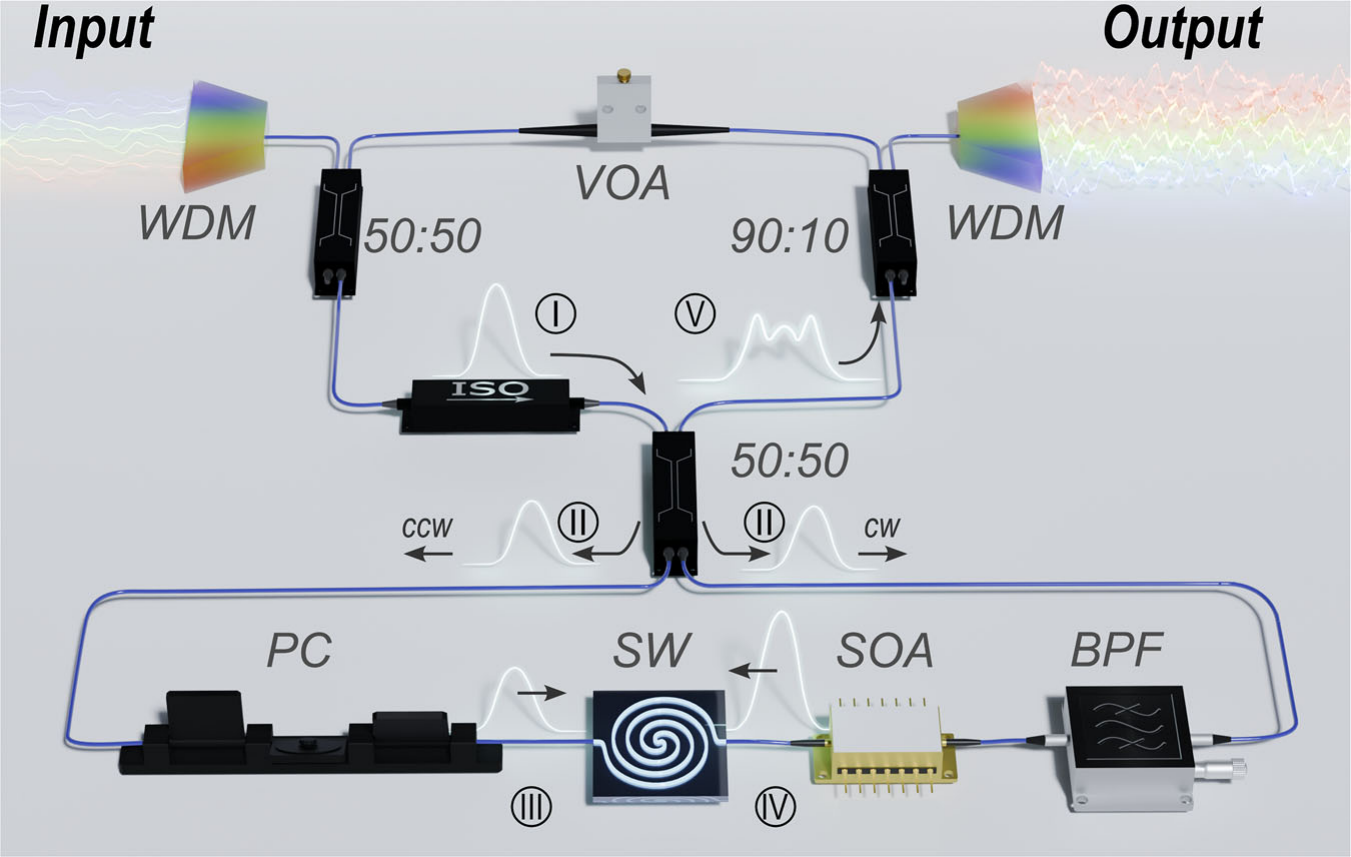
Experimental setup. The setup configuration is based on a figure-eight design, encompassing an input (top) and a processing (bottom) loop. The input loop comprises a 50:50 input optical coupler, a 90:10 output optical coupler, and a variable optical attenuator (VOA), which enables control over the intensity of the input field (I). The processing loop involves an integrated, highly doped silicon glass spiral waveguide (SW), a polarization controller (PC) that adjusts the transmission power of one of the TE or TM components, a 200 GHz bandpass filter (BPF), and a semiconductor optical amplifier (SOA). The 200 GHz filter is embedded in the loop to improve stability against the amplifier noise. The two loops are connected through a 50:50 4-port coupler, where the initial field (II) splits into two components, (III) and (IV), propagating in opposite directions. They recombine after amplification and nonlinear interaction in the same coupler. The output field (V) is demultiplexed and read out using an optical spectrum analyzer (for spectral profiles) and an ultrafast oscilloscope (for temporal profiles).

In line with the standard single-node reservoir computing framework, we establish virtual nodes within the reservoir via time-multiplexing, with training restricted to the readout layer. However, by employing multiple synchronized continuous-wave (CW) laser sources, our device can support multi-task processing across wavelength channels. This enables true concurrency for parallel data injection, both for the same or different machine learning tasks. The latter significantly reduces hardware complexity and addresses scalability and performance bottlenecks in earlier architectures. As a result, we have achieved a throughput of up to 20 tera-operations per second (TOPS), consuming only a few femtojoules per operation, while maintaining high accuracy in both classification and regression tasks. The device setup, as depicted in Fig. 1, encompasses a figure-eight fiber cavity that enables efficient multi-processing and provides rich system tunability. Such figure-eight fiber shapes can produce a range of coherent nonlinear dynamics, both with^41^ and without^42,43^ CW injection. By leveraging dynamic regimes and higher dimensionality enabled by virtual nodes, we accurately separate and classify continuous-valued input signals, even when they are complex and highly nonlinear.

## Results

We benchmarked our device to solve linearly inseparable problems using the m-bit delayed parity task, also known as the generalized exclusive OR (XOR)^16^. This nonlinear task performs the XOR operation between the current bit and the m prior bits stored in the reservoir memory and classifies the resulting sequences into two distinct classes. We injected a pseudorandom binary sequence (PRBS) of 4000 bits into a single-wavelength channel using a CW source (see the setup in Fig. 1) in the input device loop. Through the encoding procedure described in the Methods section entitled “*Device architecture*”, we generated 161 virtual processing nodes. Consistent with the RC paradigm, training was applied exclusively to the output weights, while the internal node connection weights remained fixed. These internal weights are inherently defined by the device configuration and the specific components that were employed, such as the SOA, the integrated SW, and the variable optical attenuator (VOA) (see Supplementary Note 1). We applied ridge regression^27^ to determine the optimal output weights required to reconstruct the target signal. The accuracy of the trained model was evaluated using standard performance metrics, such as the normalized root-mean-square error (NRMSE).

**Fig. 2:**
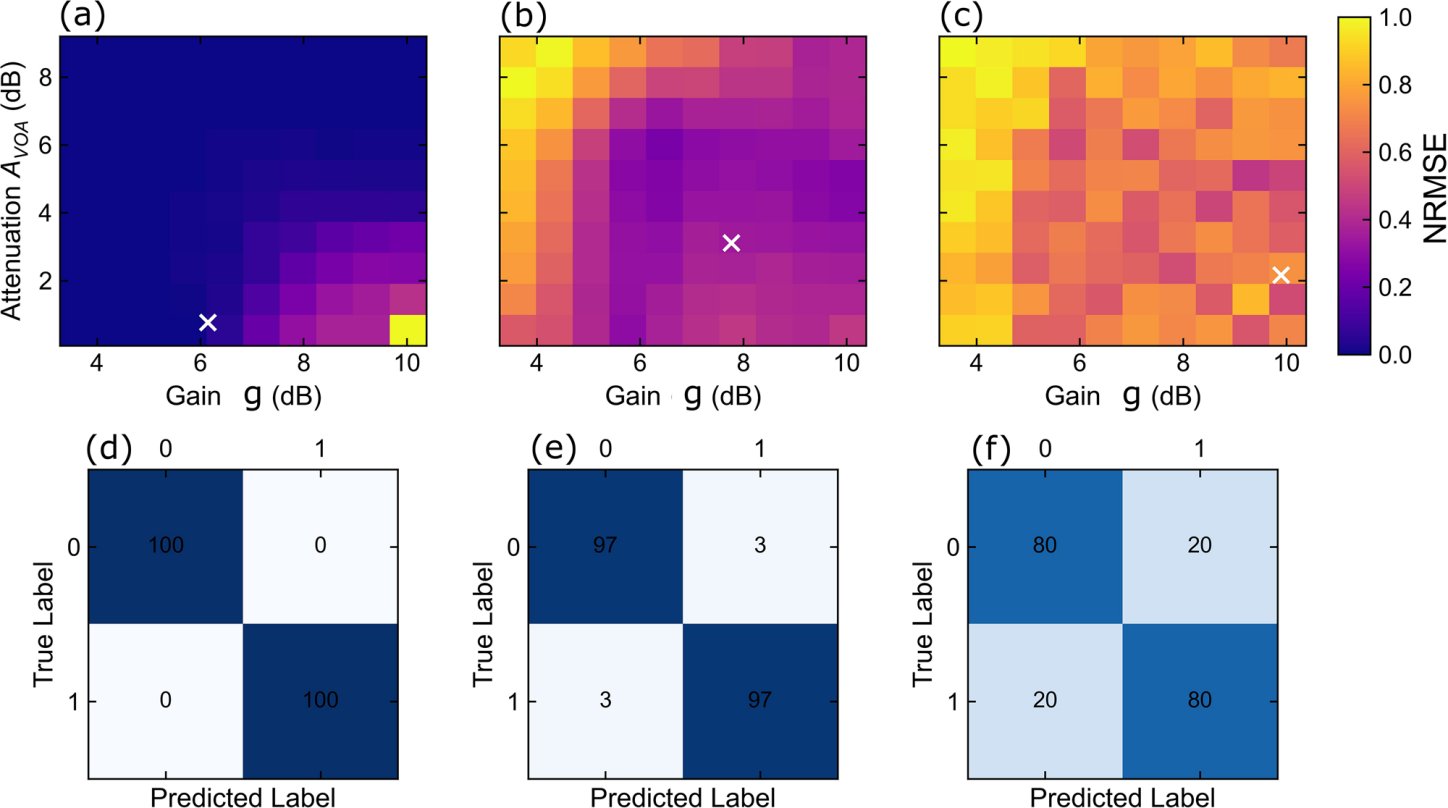
Accuracy maps for nonlinear processing optimization. **a**–**c** Parameter maps as functions of the SOA gain $(g)$ and VOA transmission $({{{\rm{A}}}}_{{{\rm{VOA}}}})$, showing the calculated NRMSE after offline training for the 2-, 3-, and 4-bit delayed XOR tasks. The attenuation of the input signals is achieved by increasing the VOA voltage, allowing control of their attenuation. The nonlinear reservoir response is modulated by the SOA gain via the driving voltage ${V}_{{{\rm{SOA}}}}$. Below each map (**d**–**f**), the corresponding confusion diagrams provide a visual representation of the processing performance. The parameters considered for the diagrams are $g=[6.1{\mbox{dB}},7.8{\mathrm{dB}},9.9{\mbox{dB}}]({V}_{{\mathrm{SOA}}}=[0.5{\mbox{V}},0.7{\mbox{V}},1.3{\mbox{V}}])$ and ${A}_{{\mathrm{VOA}}}=[0.8{\mbox{dB}},3.1{\mathrm{dB}},2.1{\mbox{dB}}]$ $({V}_{{{\rm{VOA}}}}=[2{\mbox{V}},2.7{\mbox{V}},2.5{\mbox{V}}])$ for *n* = 2, 3, 4, respectively.

We investigated the classification performance when varying the SOA gain and the signal attenuation of the VOA by tuning their driving voltages, ${V}_{{\mathrm{SOA}}}$ and ${V}_{{\mathrm{VOA}}}$, respectively (which correspond to a specific level of amplification gain $g$ and attenuation level ${A}_{{\mathrm{VOA}}}$). Figure 2 shows the NRMSE maps ($g$ and ${A}_{{\mathrm{VOA}}}$) for 2-bit, 3-bit, and 4-bit delayed sequences in (a), (b), and (c), respectively. For the 2-bit XOR, we achieved 100% classification accuracy for the validation dataset, as shown in the dark-blue shaded region of Fig. 2a, which is defined by $g < 8.3{{\rm{dB}}}$ and ${A}_{{\mathrm{VOA}}} < 4.9 \, {{\mathrm{dB}}} \left({V}_{{\mathrm{SOA}}} \right. < 0.8 \, {{\mbox{V}}}$ and ${V}_{{{\rm{VOA}}}} > 3{{\rm{V}}}$). These values delimit operational regimes characterized by reduced nonlinear contributions from the SOA and by short fading memory controlled by the VOA.

For the 3- and 4-bit XOR sequences, a further nonlinear contribution to the overall response (i.e., the activation function) of the reservoir is required to enhance the output separability and reduce the NRMSE^16^. This is achieved when setting $g > 6.1{{\rm{dB}}}$ and ${A}_{{\mathrm{VOA}}} > 0.3{\mathrm{dB}}$ (${V}_{{{\rm{SOA}}}} > 0.5{{\rm{V}}}$ and ${V}_{{\mathrm{VOA}}} > 1.5{{\rm{V}}}$), respectively, for the 3- and 4-bit cases. This enables the device to achieve accuracies of 97% and 80% (with NRMSEs of 3% and 20%), as reported in Fig. 2b and c.

Reservoir tunability enables handling ML tasks with varying degrees of data separability and temporal correlations, as evident in the analysis of the effect on the propagating input signals as a function of the two tunable parameters. When an input signal, such as an optical field, enters the device reservoir loop through the 3 dB (50:50) input beamsplitter (Fig. 1), it separates into propagating and counter-propagating field components. Their phase difference, after they propagate in opposite directions and recombine at the beamsplitter, is affected by sequences of amplification and nonlinear interactions mediated by the intensity-dependent Kerr nonlinearity in the integrated SW^52,53^. During such an interaction, an increase in the amplification gain causes the SOA to contribute additional nonlinear responses, thereby enhancing classification performance when further nonlinear contributions are required^54^, without altering the physical reservoir topology. This unsaturated operational regime enables us to implement a controllable mechanism mediated by tunable parameters, allowing for the exploitation of nonlinearities arising from controlled optical feedback and intensity-dependent interference within the NALM. This approach allows for precise and reconfigurable reservoir functionality. In contrast to the cross-gain saturation-based nonlinearities demonstrated in earlier works^50,51^. Our architecture provides greater flexibility and control over reservoir dynamics. Generally, in time-delayed RC, a higher reservoir nonlinearity can impair the fading memory, which is determined by the system’s round-trip time^46,55^. By fine-tuning the input intensity attenuation through ${V}_{{\mathrm{VOA}}}$, we can modify the strength of the interconnection among the reservoir virtual nodes. This adjustment regulates the amount of circulating intra-cavity power per round-trip, thereby compensating for the reservoir’s diminished capacity to store bits within its fading memory^56^.

### Multi-processing and parallel operation

We established the multi-processing capabilities of our device by carrying out two well-known ML benchmarks in parallel: the Mackey-Glass (MG) and the 10th-order Nonlinear Auto Regressive Moving Average (NARMA_10_) time series prediction tasks. These two tasks address the nonlinearity and memory of the reservoir, challenging the trade-off that occurs between them. The chaotic nature of the MG system, with high sensitivity to initial conditions, requires a reservoir with high nonlinearity. On the other hand, NARMA_10_ requires a reservoir with a long fading memory to efficiently retain temporal dependencies among data points across various round-trips. Therefore, by performing both tasks simultaneously, we demonstrate the universal multi-processing potential of our device in tackling both of these diverse ML tasks.

**Fig. 3:**
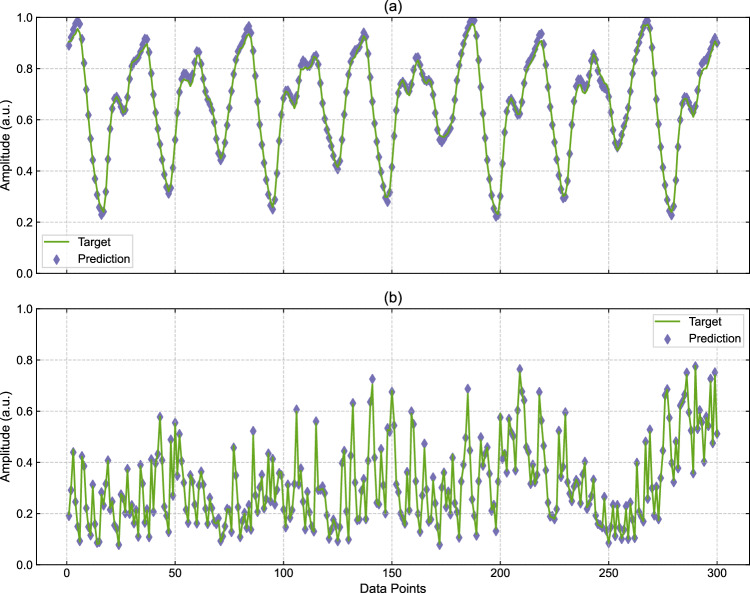
Performance results from MG and NARMA_10_ multi-processing. The figure reports the overlap between the targeted time series (green line) and the predicted output (purple diamonds) for the **a** MG and **b** NARMA_10_ tasks, which were performed in parallel. For each time series, the training results are presented for 300 points used as the validation dataset. We report the highest accuracies achieved with $g=10.3{{\rm{dB}}}$ (${V}_{{{\rm{SOA}}}}=1.5$ V) and ${{{\rm{A}}}}_{{{\rm{VOA}}}}\approx 0$ dB (${V}_{{{\rm{VOA}}}}=0.5{{\rm{V}}}$) with a 6% NRMSE for the MG task and 1% NRMSE for the NARMA_10_ task, indicating high classification accuracy for both.

We use two wavelength channels, one at 1549.2 nm for the MG task and the second at 1549.4 nm for NARMA_10_. Crosstalk effects were reduced by applying a 0.2 nm spectral separation between the channels. We generated 1200 data points for each time series and encoded them onto ${{{\rm{N}}}}_{{{\rm{v}}}}=161$ virtual nodes in the reservoir through dense WDM. Based on the results of the m-bit XOR task (where the highest accuracies are achieved within the dark-blue-shaded region shown in Fig. 2a), we found that the best performance can be reached with $g=10.3{{\rm{dB}}}$ (${V}_{{{\rm{SOA}}}}=1.5{{\rm{V}}}$) and ${A}_{{\mathrm{VOA}}}\approx 0\,{\mathrm{dB}}$ (${V}_{{{\rm{VOA}}}}=0.5{{\rm{V}}}$). We applied offline training and cross-validation^57^ independently on the two de-multiplexed outputs to obtain the weights that minimize the NRMSE between the targeted and predicted outputs. The results in Fig. 3 show misclassification rates of just 6% for MG and 1% for NARMA10, indicating high classification accuracy in both cases.

### Computational scalability of the device

We demonstrate system scalability through both increased bit density and parallel channel encoding, enabling the scaling up of computational throughput without requiring additional physical nodes. We next applied the device to solve a real-world task: recovering a nonlinearly perturbed telecom signal. Signal distortion, attenuation, dispersion, noise, and crosstalk originating from fiber propagation can all degrade or disrupt communications. Therefore, channel recovery techniques are essential for preserving signal integrity, minimizing downtime, and ensuring quality of service in networks^29,58^. This requires nonlinear, low-latency, high-speed processing capabilities. As shown by previous benchmarks, our device offers features that can meet these requirements, and its computational speed can be further enhanced by employing a dense information encoding scheme alongside multi-channel processing. The dense encoding scheme (see the Methods section entitled “*Information encoding and readout*”) enables us to inject multiple bits per wavelength channel into the reservoir, thereby increasing speed without adding extra computational units.

**Fig. 4:**
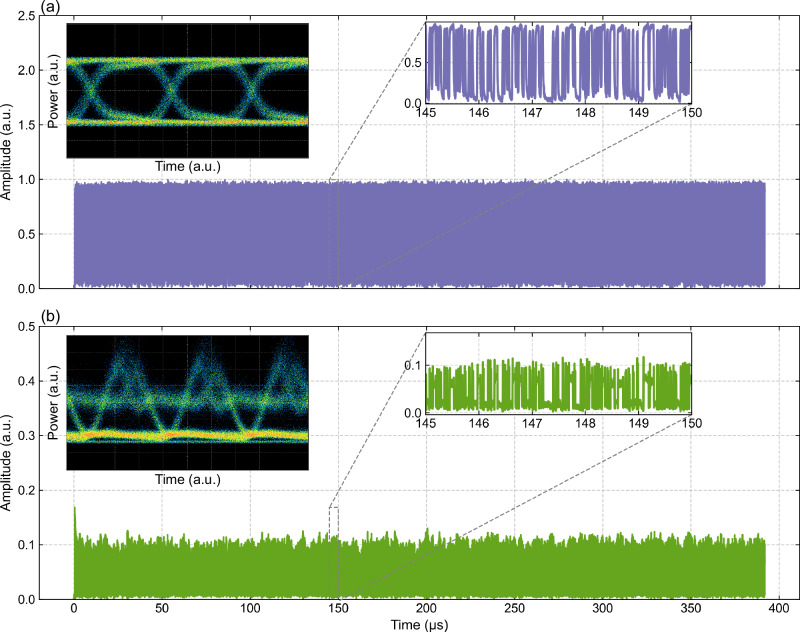
Pristine vs. distorted telecom signal. **a** A portion of the pristine temporal trace (i.e., the original telecom signal) is encoded into the CW wavelength channel. **b** The same portion of the distorted time trace after propagation. The eye diagram in the inset of (**a**) shows a large opening, indicating a high-quality signal with good integrity, low timing jitter, and high noise margin, demonstrating that the system is capable of reliable data transmission with an initial low BER. Conversely, the eye diagram inset in (**b**), with a filled triangular shape, suggests that the system is experiencing signal degradation, resulting in a decreased BER.

We generated a PRBS of 5 × (2¹⁵ − 1) bits and electro-optically encoded it onto a CW carrier signal. We then let the signal propagate through 60 km of standard single-mode fiber (SMF). Here, the once pristine signal experiences dispersion and nonlinear distortion, including cross-phase modulation and four-wave mixing, both of which are influenced by fiber length and signal intensity. Figure 4a and b show the time traces of the modulated field intensity using PAM^30,31^ before and after propagation, respectively, along with their corresponding eye diagrams. The distorted signal is then distributed and encoded across five independent channels, spaced at 0.2 nm intervals around 1550 nm (as shown in Fig. 4b), which we process concurrently via dense WDM. The number of encoding channels is limited only by the available CW sources and electro-optical intensity modulators (IMs). The representative bit sequences before and after recovery are shown in the Supplementary Fig. 5.

**Fig. 5:**
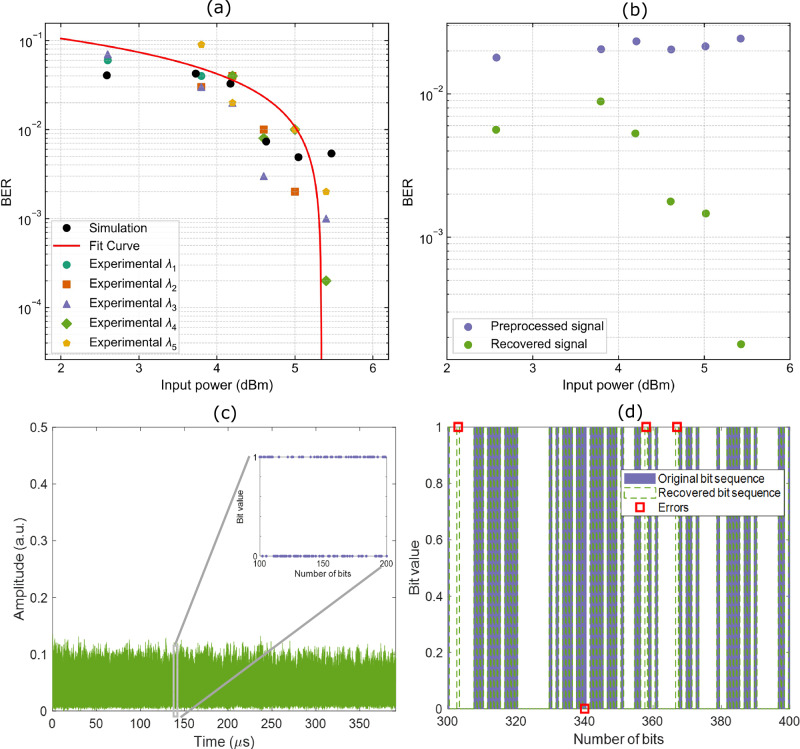
BERs of the five channels as a function of input power. **a** BER of all five channels (which are indicated by five different markers) after processing and ML-assisted reconstruction. The black dots denote the simulated values, which are compared with the average BER across the five parallel wavelength channels. A good agreement between the simulation and experimental results validates our theoretical model. **b** BER values of the distorted signal before (purple markers) and after (green markers) recovery with our device. **c** Experimentally measured distorted optical signal (green trace) after nonlinear propagation through 60 km of standard single-mode fiber at 5.2 dBm input power. The inset highlights a zoomed portion of the original bit sequence, which was encoded in the modulated signal amplitude transmitted over the fiber. **d** A portion of the recovered bit sequence obtained after processing the distorted input shown in (**c**) using our trained device. The plot compares the recovered and original bit sequences, with red squares indicating the positions of bit errors. These results support the BER performance reported in (**a**, **b**) and demonstrate the device’s ability to reconstruct transmitted digital information from severely degraded optical signals.

We trained and validated the output weights for each channel across four virtual nodes using the encoding technique and offline ridge regression. The bit error rate (BER, the metric that quantifies the percentage of misclassified bits in the recovered sequence) is shown in Fig. 5 (a), illustrating the individual channels and different input power levels (P_in_ = 2.6 dBm, 3.8 dBm, 4.2 dBm, 4.6 dBm, 5.0 dBm, and 5.4 dBm), which are commonly used in telecommunications. We observed that the BER decreases as the input signal power increases, due to improved signal-to-noise ratio.

This change occurs regardless of the surge in distortion due to the intensity-dependent Kerr effect on transmission, which our approach can compensate for. The lowest BER value is 2 × 10^−4^ at 5.4 dBm of input power, representing a 145-fold improvement compared to the preprocessed distorted signal (see Fig. 5b). The inset of Fig. 5c displays a segment of the original bit sequence alongside the corresponding distorted optical signal, measured after propagation through the 60 km fiber link (prior to recovery processing). Figure 5d shows the recovered bit sequence at an input power of 5.4 dBm, with a zoomed-in segment highlighting the bit errors (marked with red squares).

The experimental results were validated using the model derived from the generalized nonlinear Schrodinger equation^59^ (GNLSE) (see Supplementary Note 1). We report the average BER over the five wavelength channels (red line) and the simulated BER values (black dots) in Fig. 5a. We observed excellent agreement between the numerical predictions and our experimental results, confirming the accuracy of our model.

We can quantify the throughput of our device in terms of MAC (multiply-and-accumulate) operations per second for parallel signal recovery. The device achieves a 2.7 Tera-MAC s^-1^. From the MAC calculation, we estimate a speed of 20 Tera-operations per second (TOPS) for our device. In terms of energy efficiency, the overall power consumption of the processing unit is approximately 87 mW, leading to consumption of ~ 4.4 fJ OP^-1^ (see Supplementary Note 4 for detailed calculations), which outperforms both state-of-the-art fiber-based optical neural networks and photonic chips^5,8,35,60–62^. See Table 1 for a numerical comparison.

**Table 1:** Comparison of speed and energy consumption in photonic processing devices

Work	Speed (TOPS)	Energy Consumption (pJ OP^−1^)	ML Task	Computational Accuracy
Our device	20	0.0044^a^	Nonlinear channel recovery	BER = 2 × 10^−4^
Gu et al.^62^	5.25	0.011^a^	Fashion-MNIST and object recognition	Accuracy^c^ = 90.18%
Ashtiani et al.^5^	0.27	14	Image classification	Accuracy = 93.18%
Xu et al.^8^	6.63^b^	6.22	1000-category-level classification	Accuracy = 91.89%
Bandyopadhyay et al.^60^	0.59	4.6	Vowel classification	Accuracy = 92.5%
Shen et al.^20^	0.02	6^a^	Nonlinear channel equalization	BER = 2 × 10^−3^
Lupo et al.^38^	0.004	NA ( ≈ 1000)a	Nonlinear channel equalization	BER = 1 × 10^−3^

^a^Estimated considering just the power and energy consumption of the processing unit.

^b^Estimated considering a clock frequency of 2.0 GHz and a single-chiplet as reported.

^c^Accuracy is defined as (1-NRMSE) × 100%.

## Discussion

In this work, we developed a tunable neuromorphic computing device based on an NALM configuration, thereby realizing a time-delayed RC circuit. This device was applied to the high-speed, low-latency processing of nonlinear, complex ML tasks, including classification for forecasting and regression for telecom signal recovery.

This device achieves high classification accuracy in the m-bit delayed-XOR task by optimizing reservoir parameters. It also demonstrates effective handling of parallel machine learning tasks (i.e., multitasking), as shown by its performance on the MG and NARMA_10_ time series, with a minimum prediction accuracy of more than 94%.

The reservoir’s throughput is directly linked to its fundamental clock frequency, which is defined as the inverse of the round-trip time^63,64^. In our system, this corresponds to 13.3 MHz (75 ns round-trip), which sets the device’s intrinsic throughput and, therefore, its inherent throughput. However, because the architecture supports the simultaneous processing of multiple wavelength channels, the system can scale its throughput without requiring additional physical components. Using Eq. (17) reported in the Supplementary Note 4.1, we calculated the device throughput for telecom signal recovery, obtaining a value of 20 TOPS and a minimum BER of 2 × 10⁻⁴ for the recovered signal.

Advanced digital signal processing (DSP) methods, including digital backpropagation^64^, typically report BER values around 2 × 10⁻². Real-time field-programmable gate array-based coherent receivers have achieved 2.4 × 10⁻⁴ at 15 Gbaud with 16-quadrature amplitude modulation (16-QAM)^65^. These approaches require thousands of multipliers and significantly higher power consumption^64,66^, typically around 3 pJ per bit per channel^67,68^, with latency between 100 ns and 1 μs, depending on the system clock^69^. In contrast, optical signal processing methods offer a latency of 20 to 200 ps and energy consumption ranging from 0.1 to 1 pJ per bit per channel^70,71^.

In terms of signal quality enhancement achieved after compensation, electronic neural networks^72^ can reach a Q-factor improvement of 2 dB with a BER of about 1.1 × 10⁻³, while photonic–electronic hybrid networks^73^ can achieve an improvement of about 0.8 dB with a BER around 10^−4^.

Our device achieves a BER of 2 × 10⁻⁴ after 60 km of transmission at an input power of 5.4 dBm, corresponding to a Q-factor improvement of approximately 4.6 dB (see Fig. 5b). It operates at 4.4 fJ per operation, outperforming DSP and electronic neural-network processing as well as previously reported photonic neural-network implementations, as summarized in Table 1.

This performance enhancement stems from our reservoir architecture, which primarily relies on integrated passive and low-power active elements (see Supplementary Note 4.2). The design enables parallel processing with concurrent encoding, thus reducing peripheral overhead, eliminating redundant computational layers, and minimizing iterative losses.

Unlike conventional reservoir architectures that rely on sequential or interleaved data injection, our approach processes all wavelength channels concurrently within a single unit, thus enabling true parallelism. Combined with dense encoding, this removes the latency bottleneck and allows throughput to scale directly with the number of available channels. However, the maximum processing speed is constrained by the intrinsic carrier dynamics of the SOA and by the operational boundaries set to avoid saturation, which together define the highest achievable computational throughput. Under our operating conditions, and using the equation derived in Supplementary Note 4.1 (Eq. 21), the maximum distortion-free throughput is theoretically estimated at 24 TOPS per channel. It must be noted that while the architecture can support this level of throughput, its practical performance is lower due to the limited bandwidth of the encoding hardware.

Further scalability in processing can be achieved through dense wavelength-division multiplexing (DWDM), which provides the spectral density required for massively parallel operation. For instance, with 80 channels, as is typically achieved in standard DWDM, the architecture can reach a combined throughput of nearly two peta-operations per second (POPS) under binary modulation. By employing phase-sensitive processing and higher-order modulation formats, such as 64-quadrature-amplitude modulation (6 bits per symbol), the effective throughput can increase to several tens of POPS. Integrated multi-wavelength sources based on nonlinear processes such as four-wave mixing in microresonators enable this capacity by supplying many stable, phase-coherent channels on a single chip^74–78^.

These findings open new avenues for the development of affordable, scalable neuromorphic platforms to address the growing demand for ultra-fast, energy-efficient solutions in next-generation photonic signal processing and AI applications.

## Methods

### Device architecture

The device’s figure-eight cavity design (see Fig. 1) can be divided into input, processing (i.e., the reservoir or unit), and output stages, all of which contribute to the overall round-trip time and thereby cause the reservoir’s memory to fade. The input loop comprises a variable optical VOA (Thorlabs, V1550A) that controls the signal power, a 10:90 coupler that monitors the system, a 50:50 coupler that injects the modulated signal, and a second coupler that splits the signals into clockwise and counterclockwise directions in the NALM configuration. The NALM loop is composed of an SOA (Inphenix C-Band IPSAD1505) and an integrated 45 cm-long SW. This waveguide is fabricated from a CMOS-compatible, highly doped silica glass^52,53,78–83^. As such, the SW features high mode confinement due to the small waveguide profile (2 × 2 µm^2^), with group velocity dispersion coefficients of ${\beta }_{2}$ ∼ 10 ps^2^ km^−1^ and ${\beta }_{3}$ ~ 0.26 ps^3^ km^−1^ at 1550 nm, as well as a large third-order nonlinear coefficient of *γ* = 220 W^−1^ km^−1^. The SW, together with the phase shift mechanism between the two counter-propagating fields, performs the overall nonlinear activation function of the reservoir^52,53^. The SOA operates between the linear and nonlinear regimes, far from saturation, to avoid instabilities. These include self-oscillation and chaotic behaviors, as well as noise amplification and signal fluctuations, which would be detrimental to the current tasks. A polarization controller (PC) was used to select and optimize the transmission power of one field component, specifically either the transverse electromagnetic or magnetic field. Simultaneously, an optical bandpass filter (200 GHz) was used to limit the spectral bandwidth of the propagation fields, thereby improving the signal-to-noise ratio (SNR) and system stability by eliminating unwanted amplified spectral components. A 90:10 optical coupler is used to extract a fraction of the optical power from the loop to obtain a readout of the dynamic evolution of the node states, utilizing high-bandwidth photodiodes (PDs, Finisar XPDV21x0R series, 50 GHz bandwidth) and a digital oscilloscope (OS, Agilent DSO-x92804a, 28 GHz bandwidth). An additional OS is employed to perform the nonlinear recovery task. The overall length of the cavity is 14 m, corresponding to a round-trip time of approximately ${\tau }_{r}=75{{\rm{ns}}}$.

### Information encoding and readout

We encode information (i.e., bits) in time-dependent vectors ${{{\rm{u}}}}_{{{\rm{i}}}}\left({{\rm{n}}}\right)$ by means of different arbitrary waveform generators (AWGs) (Keysight M8196A, 92 GSa s^-1^, 2 channels each), each driving an equal number of electro-optical Mach-Zehnder intensity modulators (IMs) (Optilab IML-1550-40-PM-V-HER, 40 GHz, polarization-maintaining, high extinction ratio, ${V}_{\pi }$ = 3 V). All modulators were synchronized using an external clock. The IMs were excited by the time-varying voltage signal containing the data, which in turn modulated the independent laser modes (i.e., the wavelength channels) produced by a narrowband continuous multi-wavelength (CW) source (ID Photonics CoBrite DX G, 4 outputs, linewidth <100 kHz). An additional CW source (Tunics T100S-HP, single output, linewidth <5 MHz) was employed to provide the fifth channel. The injected power per channel ranged from 1 mW to 5 mW, depending on the task. The applied voltage to the IMs varied between [-${V}_{\pi }/2$, ${V}_{\pi }/2$], ensuring operation within their linear regime. When multi-processing is performed, the input channels are injected into the device via wavelength division multiplexing (WDM) using a waveshaper (WS).

The reservoir nodes are established by first sampling and holding the input signal containing data for a duration $\tau \approx {\tau }_{r}$, then multiplying the signal by a uniformly distributed, multi-level random mask $R\left(n\right)$, with values ranging between −1 and 1. The mask has a sampling period ${{\rm{\tau }}}$ and duration ${{\rm{\theta }}}$ per level, such that ${E}_{{{\rm{in}}}}\left(n\right)=u\left(n\right)\cdot R\left(n\right)$, where ${E}_{{{\rm{in}}}}\left(n\right)$ is the resulting input signal to the device. This procedure maps the signal onto a higher-dimensional space by generating a number of ‘virtual’ node states (i.e., internal variables)^84^, the number of which is given by ${N}_{v}=\tau /\theta$. A slight desynchronization, $\delta \approx \theta$, was applied to the sampling period $\tau$ on the AWG input sequence to ensure that all node states were dependent on neighboring nodes^17^.

For dense encoding, we input more than one bit of information (${n}_{{{\rm{b}}}}$) in the reservoir per round-trip ${{{\rm{\tau }}}}_{{{\rm{r}}}}$. We apply the sample and hold procedure over the asynchronous sampling rate (i.e., the inverse of the masking rate) of duration ${{\tau }_{{{\rm{r}}}}}^{{\prime} }={\tau }_{{{\rm{r}}}}/{n}_{{{\rm{b}}}}+\delta {\prime}$ to generate $N\hbox{'}$ time-multiplexed transient states of duration ${\theta }^{{\prime} }={{\tau }_{{{\rm{r}}}}}^{{\prime} }/{N}_{v}^{{\prime} }$, for each ${{\tau }_{r}}^{{\prime} }$ time interval.

The small desynchronization $\delta {\prime}$ $\approx \theta {\prime}$ between the sampling and injection rates causes a progressive shift in the input seen by adjacent virtual nodes and by nodes across round-trips, ensuring that neighboring nodes access distinct input segments over time and maintain the data correlation required for reservoir computing processing.

The output states, ${x}_{i}\left(n\right)$, are measured from the virtual nodes at time intervals $\theta$ (or ${\theta }^{{\prime} }$) by using the PDs and OS. De-multiplexing using the WS is applied in cases of parallel computations. The average output power ranges from 100 μW to 300 μW per individual input channel.

### M-bit delayed XOR task

To formally describe the m-bit delayed XOR task^85^, we define the operation within a logical framework. Given the input bit sequence ${\{x(n)\}}_{m}$ with delay $n$ and the number of bits $m$, the output sequence ${\{{y}_{n}\}}_{m}$ can be described by the following equation:

1 $$\left\{\begin{array}{c}{y}_{n}={x}_{n}\oplus {x}_{n-1}\oplus {x}_{n-2}\oplus \ldots \oplus {x}_{n-m}{{\mbox{if}}} n\ge m\\ {y}_{n}={x}_{n}{{\rm{if}}}n < m \hfill\end{array}\right.$$

where ${x}_{n}$ and ${y}_{n}$ are the current and output bits at time ${{\rm{n}}}$, respectively.

### Time series prediction tasks

The Mackey-Glass (MG) time series is defined by the following equation:

2 $${\dot{y}}_{{\mathrm{MG}}}\left(t\right)=\beta \frac{{y}_{{\mathrm{MG}}}\left(t-\tau \right)}{1+{y}_{{\mathrm{MG}}}^{p}\left(t-\tau \right)}-h{y}_{{\mathrm{MG}}}\left(t\right)$$

in which ${y}_{{{\rm{MG}}}}(t)$ is the output at a time $t$, $\tau$ is the delay time, $\beta$ is the growth rate, $h$ is the decay rate, and $p$ is the nonlinear parameter. Parameters were set as follows: $\tau=17,\beta=0.2,h=0.1,p=10$. To simulate MG time series behavior, ${{\rm{t}}}$ was discretized using a time step $\Delta t=1$ to produce values $x(t\equiv {t}_{k})$ at discrete time points ${t}_{k}=q\Delta {{\rm{t}}}$ for $q=0,1,2,\ldots$.

The 10th-order Nonlinear Auto Regressive Moving Average (NARMA_10_) series follows the equation

3 $${y}_{N10}\left(t+1\right)=0.3y\left(t\right)+0.05y\left(t\right){\sum}_{i=0}^{9}y\left(t-i\right)+1.5u\left(t\right)x\left(t-9\right)+0.1$$

in which$\,{y}_{N10}\,\left(t\right)$ is the output at time $t$, ${{\rm{while}}}$ $u(t)$ is the input, chosen as a random sequence from a uniform distribution ${{\rm{U}}}[0,1]$. Similarly to the MG task, we applied a discretized time step $\Delta t=1$ to the NARMA_10_ time series temporal variable $t$.

The time series were simultaneously and independently encoded onto two wavelength channels generated by the multi-output CW source. Two IMs and AWG channels drove this process. Following the previously described single-bit encoding technique, both signals were multiplexed using the WS and then injected simultaneously into the device. They were then de-multiplexed and read with the OS. Offline training (as detailed in the following sections) was performed on the two datasets collected.

### Nonlinear telecom signal recovery task

The nonlinearly distorted signal, which also carries information, was obtained by propagating the corresponding pristine signal through 60 km of SMF-28, with a dispersion parameter *D* = 18 ps nm^−1^ km^−1^ and an attenuation constant *α* = 0.24 dB km^−1^. An additional 1 m of highly nonlinear optical fiber (with *γ* = 0.78 W^−1^km^−1^) was added to account for the full extent of nonlinear effects over longer propagation distances, which is typical in telecommunications networks. The distorted signal, carrying 5 × (2^15^ – 1) bits, was sequentially split into five subsequences of 2^15^ – 1 bits each and then independently encoded into the five wavelength channels using five IMs and AWG channels. Each AWG channel was operated at ~6.5 GHz. All five channels were amplified to 5 mW using an erbium-doped fiber amplifier (EDFA, Keopsys, PEFA-SP-C-PM−27-B202), then multiplexed into the reservoir. The additional IM and AWG channel were used to create the temporal node transients ${\theta }^{{\prime} }$ via time multiplexing. These were implemented at a masking rate of approximately 24 GHz, creating 4 virtual nodes with a 20-level random mask, thereby enabling the encoding of 488 bits per round-trip. Offline training was performed on datasets built from the output node states of all five de-multiplexed wavelength channels, using the training procedure described in the following section.

### Offline training

We employed a k-fold cross-validation approach, with k set to 5 for the MG and NARMA_10_ tasks and to 10 for the nonlinear channel equalization task. We then split the output datasets, consisting of ${x}_{i}$ values, into equally sized *k* subsets. The machine learning (ML) model was trained using ridge regression on $k-1$ folds and validated on the remaining fold. This process was repeated k times, with each fold used once as the validation set. For each fold, we determined the model’s accuracy by calculating the normalized root mean square error (NRMSE) as

4 $${{\rm{NRMSE}}}=\sqrt{\frac{1}{{N}_{v}}{\frac{{\sum }_{i}^{{N}_{v}}\left({y{\prime} }_{i}-{y}_{i}\right)}{{\sigma }^{2}\left({y}_{i}\right)}}^{2}},$$

in which ${\sigma }^{2}$ is the sample variance, ${y{\prime} }_{i}$ is the targeted output, and ${y}_{i}$ is the weighted output: ${y}_{i}={\sum }_{1}^{{N}_{v}}{W}_{{ij}}^{{{\rm{out}}}}{x}_{j}$.

Finally, we calculated the average error across all iterations to obtain a more robust estimate of the model’s overall performance.

### Simulations for nonlinear channel equalization

We employed a 10,000-pseudorandom binary sequence (PRBS) extracted from the experimentally generated distorted signal. We then sampled the signal using ultra-dense encoding, mirroring the experimental procedure previously described. We injected 488 bits per cavity round-trip over four nodes into a single channel. The device response was numerically simulated using the generalized nonlinear Schrödinger equation (GNLSE) and then implemented in MATLAB (see Supplementary Note 1).

## Supplementary information

Supplementary Information

Transparent Peer Review file

## Data Availability

The data supporting the findings of this work study are available both within this article and in the Supplementary Information. The raw data generated from this study is available from the corresponding authors upon request.

## Code availability

The numerical codes related to this study are available from the corresponding author upon request.
