# Supplementary material for: Scalable photonic reservoir computing for parallel machine learning tasks
Source: Nat Commun. 2025 Dec 31;17:1225. doi: 10.1038/s41467-025-67983-z (PMC12864733; doi:10.1038/s41467-025-67983-z)
Supplement: Supplementary file 1 — Supplementary Information [file 41467_2025_67983_MOESM1_ESM.pdf]

Supplementary Information

Scalable Photonic Reservoir Computing for Parallel Machine Learning Tasks

A. Aadhi[1], L. Di Lauro[1*], B. Fischer[1,2], P. Dmitriev[1], I. Alamgir[1], C. Mazoukh[1], N. Perron[1], E. A. Viktorov[3], A. V. Kovalev[3], A. Eshaghi[4], S. Vakili[1], M. Chemnitz[1,2], P. Roztocki[1], B. E. Little[5], S. T. Chu[6], D. J. Moss[7], and R. Morandotti[1†]

[1]Institut National de la Recherche Scientifique - Énergie Matériaux Télécommunications; Varennes, J3X 1P7, Canada.
[2]Leibniz Institute of Photonic Technology; Jena, 07745, Germany.
[3]ITMO University; St. Petersburg, 199034, Russia.
[4]Huawei Technologies Canada; Markham, L3R 5A4, Canada.
[5]QXP Technology Inc.; Xi'an, 710119, China.
[6]City University of Hong Kong; Hong Kong, China.
[7]Optical Sciences Centre, Swinburne University of Technology; Hawthorn, 3122, Australia.

Corresponding authors. Emails: [*]Luigi.DiLauro@inrs.ca; [†]Roberto.Morandotti@inrs.ca

Supplementary Note 1. Theoretical modeling

The concept of reservoir computing (RC) is built upon two fundamental elements[1,2]: i) nonlinearity, which is responsible for introducing transformations that enable mapping of the input data points into a higher-dimensional phase space, and ii) recurrency, which establishes a short-term memory (or fading memory), preserving time-correlation among input data points within the reservoir as time progresses[2–4]. The evolution of reservoir node states $\mathbf{x}(n)$, at each discretized time n, is governed by the following general equation:

$$\mathbf{x}(n) = \mathbf{f}\,(W_{in} \cdot \mathbf{u}_{\mathrm{in}} + W_R \cdot \boldsymbol{x}(n-1) + \mathbf{b}), \tag{1}$$

where $\mathbf{u}_{\mathrm{in}} \epsilon \mathbb{C}_{\mathrm{l}}$ is the vector containing an l number of input data points, $\mathbf{f}$ is the function that characterizes the response of the reservoir, $\mathrm{W}_{\mathrm{in}} \epsilon \mathbb{C}_{\mathrm{l,m}}$ are the input weights, defined by the random matrix established through the encoding procedure (see the Methods section in the main manuscript entitled "*Information encoding and readout"*), $\mathrm{W}_{\mathrm{R}} \epsilon \mathbb{C}_{\mathrm{m,m}}$ are the fixed reservoir interconnection weights, and $\mathbf{b}\ \epsilon \mathbb{R}_{\mathrm{m}}$represents fixed bias values that can modulate the sensitivity of the virtual nodes to different input data. The processed output from the reservoir is then obtained as a weighted superposition of the node state outputs:

$$\boldsymbol{y}_{out}(n) = W_{out} \cdot \boldsymbol{f}_{out}\big(\boldsymbol{x}(n)\big), \tag{2}$$

in which $\mathbf{y}_{\mathrm{out}} \epsilon \mathbb{C}_{\mathrm{k}}$ is the vector containing the processed output values, $\mathrm{W}_{\mathrm{out}} \epsilon \mathbb{C}_{\mathrm{m,k}}$ are the readout weights that project the high-dimensional representations generated by the reservoir to the specific output values. In the RC framework, only the output weights are trained for the ML task to be solved. The function $\mathbf{f}_{\mathrm{out}}$ accounts for the transformation of the output node states due to physical measurements (e.g., photodetector nonlinearity or output noise).

Previously, it was shown that temporal nonlinear states subject to external feedback can be treated as virtual nodes of a reservoir[5]. By transitioning to a single-node time-delayed approach, Eq. (1) can be replaced by the following delayed equation:

$$x(t) = f\big(s_{\mathrm{in}} \cdot u_{\mathrm{in}}(t), s_{\mathrm{R}} \cdot x(t-\tau_{\mathrm{r}})\big), \tag{3}$$

where τ_{r} is the reservoir round-trip time, while s_{in} and s_{R} are related to the weights through scaling factors. The latter can be, in turn, linked to the tunable parameters of the reservoir by modeling the evolution of the electric field envelope E within the framework of the GNLSE[6,7]:

$$\partial_z E(z,t) = \left(\frac{1}{m!}\sum_{i=1}^{m}(-i)^m \beta_m\, \partial_t^m - \frac{\alpha}{2} + \frac{G(z,G_{\mathrm{s}})}{2} + i\gamma|E|^2\right)E(z,t), \tag{4}$$

in which $E(z,t)$ is the longitudinal component of the device propagating an electrical field, β_m represents the mth order dispersion coefficients of the waveguide, while α quantifies the propagation losses. The function G in Eq. (4) describes the amplifier gain dynamics as a function of the longitudinal field coordinate z within the gain material and the SOA drive voltage V_{SOA} as applied during the experiments, capturing both the linear regime at low voltages and the nonlinear regime at high voltages. As our experiments are carried out below the SOA saturation regime, the gain function G is obtained from the model developed by Connelly et al.[8], which accounts for the physical processes inside InP–InGaAsP SOAs (i.e., carrier density dynamics, spontaneous emission, and stimulated emission), by providing the SOA drive voltage as input. In the case of operation within the saturation regime, the gain function can be approximated as $G \approx g \cdot L$ where g is the SOA amplification gain in decibels and L is the length of the SOA active region. γ represents the third-order (Kerr) nonlinear coefficient of the integrated spiral waveguide (SW), while t is the propagation time.

With a single CW in the configuration illustrated in Supplementary Fig. 1 (a), implementing the RC-based architecture depicted in (b), the propagation of the optical field E_{in} through the different sections and components of the setup can be modeled by the following GNLSE differential operators:

$$\hat{\mathrm{F}}_{\mathrm{f}}: \partial_z + i\frac{\beta_2^{\mathrm{fb}}}{2}\partial_{tt} + \frac{\alpha_{\mathrm{fb}}}{2}, \tag{5}$$

$$\hat{\mathrm{F}}_{\mathrm{SW}}^{\gamma}: \partial_z + i\frac{\beta_2^{\mathrm{SW}}}{2}\partial_{tt} - \frac{\beta_3^{\mathrm{SW}}}{6}\partial_{ttt} + \frac{\alpha_{\mathrm{SW}}}{2} - i\gamma\left|E_2^{\pm}\right|^2, \tag{6}$$

$$\hat{\mathrm{F}}_{\mathrm{G}}: \partial_z + i\frac{\beta_2^{\mathrm{fb}}}{2}\partial_{tt} + G, \tag{7}$$

in which β_2^{fb} and β_2^{SW} represent the group velocity dispersion of the fiber and SW, respectively, while β_3^{SW} accounts for the third-order dispersion coefficient of the SW material. α_{fb} and α_{SW} are the loss coefficients. If the 50:50 bidirectional coupler in the NALM has a negligible length, we can obtain the clockwise and counterclockwise field expressions by means of the following approximation:

$$E_1^{+} = \sqrt{k}E, \tag{8}$$

$$E_1^{-} = -i\big(\sqrt{1-k}\big)E, \tag{9}$$

where k is the coupling ratio, while $E_1^{\pm}$ are the fields entering the NALM section before interacting with the SW and SOA. E is the fraction of the input field E_{in}, injected through the 50:50 input coupler as $E = \sqrt{k}_{\mathrm{in}}E_{\mathrm{in}}$, where k_{in} is the coupling ratio of the input coupler.

By applying the operators given by Eqs. (6) and (7) onto the fields $E_1^{\pm}$, the NALM output fields can be written (after propagating in the SW and SOA) as:

$$E_2^{+} = \hat{\mathrm{F}}_{\mathrm{G}}\hat{\mathrm{F}}_{\mathrm{SW}}^{\gamma}\, E_1^{+}, \tag{10}$$

$$E_2^{-} = \hat{\mathrm{F}}_{\mathrm{SW}}^{\gamma}\hat{\mathrm{F}}_{\mathrm{G}}\, E_1^{-}, \tag{11}$$

The overall recombined field from the NALM coupler output, E_{NALM}, can be the expressed as:

$$E_{\mathrm{NALM}} = -i\left(\sqrt{1-k}\right)E_2^{-} + \sqrt{k}E_2^{+}, \tag{12}$$

Since the reservoir operates in a delayed configuration (closed figure-eight fiber loop), the field E(t) is determined by the superposition of the injected field $\mathrm{E}_{\mathrm{in}}(\mathrm{t})$ and the propagating NALM field, $\mathrm{E}_{\mathrm{NALM}}(t-\tau_r)$, which is also considered the reservoir field from the previous round-trip at the time $t-\tau_r$. The following recurrent condition expresses this relationship:

$$E(z,t) = \sqrt{k_{\mathrm{in}}}E_{\mathrm{in}}(t) + i\sqrt{1-k_{\mathrm{in}}}\sqrt{k_{\mathrm{VOA}}}\sqrt{1-k_{\mathrm{out}}}\hat{\mathrm{F}}_{\mathrm{f}}E_{NALM}(z,t-\tau_{\mathrm{r}}), \tag{13}$$

in which k_{in} and k_{out} represent the self-coupling coefficients of the input and output couplers, respectively (see Supplementary Fig. 1). The effect of the VOA on optical field attenuation is linearly mapped to the dimensionless parameter k_{VOA}, which ranges from 0 (full attenuation) to 1 (no attenuation), according to the data provided by the manufacturer (see the Methods section in the main manuscript entitled –"*Device architecture"*).
By combining Eqs. (8-11) into Eq. (12), $\mathrm{E}_{\mathrm{NALM}}(z,t)$ can be explicitly expressed as:

$$E_{\mathrm{NALM}}(z,t) = -i\left(\sqrt{1-k}\right)\hat{\mathrm{F}}_{\mathrm{SW}}^{\gamma}\hat{\mathrm{F}}_{\mathrm{G}}\left[-i\left(\sqrt{1-k}\right)E(z,t)\right] + \sqrt{k}\hat{\mathrm{F}}_{\mathrm{G}}\hat{\mathrm{F}}_{\mathrm{SW}}^{\gamma}\sqrt{k}E(z,t), \tag{14}$$

By comparing Eq. (13) with Eq. (3) and then using Eq. (14), we infer that $s_{\mathrm{in}} = \mathrm{i}\sqrt{k_{\mathrm{in}}}\mathrm{W}_{\mathrm{in}}$ and $s_R = ik_{VOA}(V_{VOA})W_R\left(1-\sqrt{k}\right)\sqrt{k}$. From the expression of s_{R}, it can be observed that the strength of the internal connections (i.e., the fading memory) is modulated by the VOA attenuation voltage V_{VOA}, which multiplies the internal reservoir weights. The nonlinear behavior of the reservoir is influenced by the nonlinear amplification response of the SOA through its driving voltage,$\mathrm{V}_{\mathrm{SOA}}$, which modifies the overall nonlinear phase shift experienced by the fields, as indicated by Eq. (14). In our case, the bias b can be attributed to polarization effects, asymmetric splitter coupling ratios, and noise. Finally, the measured device output field is given by:

$$E_{\mathrm{out}} = -i\sqrt{k}_{\mathrm{out}}E_{\mathrm{NALM}}, \tag{15}$$

All parameter values are reported in Supplementary Table 1.

Supplementary Note 2. Reservoir tunability

Using the model derived in Supplementary Note 1, we simulate the device response to a train of modulated super-Gaussian pulses generated using the single-bit data-encoding procedure described in the Methods. Such pulses have a linewidth of 466 ps (equivalent to spacing between 161 virtual nodes embedded in a $\tau_{\mathrm{r}} = 75$ ns feedback loop) and a repetition rate equal to $1/\tau_{\mathrm{r}}$.
In Supplementary Fig. 2, we plot the amplitude of the output field $\mathrm{E}_{\mathrm{out}}$ for the values of gain, $g = 6.1$ dB ($\mathrm{V}_{\mathrm{SOA}} = 0.5$ V) and $g = 10.3$ dB (1.5 V) (top (a-c) and bottom (d-f) of Supplementary Fig. 2, respectively), with attenuation values increasing from $\mathrm{A}_{\mathrm{VOA}} \approx 0$ dB ($\mathrm{V}_{\mathrm{VOA}}$= 0.5 V) in (a) and (d) to $\mathrm{A}_{\mathrm{VOA}} =$ 1 dB ($\mathrm{V}_{\mathrm{VOA}}$= 2.1 V) in (b) and (e), continuing to $\mathrm{A}_{\mathrm{VOA}} = 5.2\ dB$ ($\mathrm{V}_{\mathrm{VOA}}$= 3 V) in (c) and (f). For $g =$ 6.1 dB, we observe that propagating pulses fade after fewer round-trips as the VOA attenuation increases

(i.e., when increasing the parameter V_{VOA}). However, when attenuation allows for sufficient fading memory, low-intensity signal components persist in the reservoir feedback loop, overlapping with signals injected in subsequent round-trips. This results in higher-intensity peaks sustained by SOA amplification, allowing for interactions between virtual nodes across time and preserving temporal correlations among input data points. In contrast, we observe decreased overlap among consecutive pulses at $g = 10.3$, despite the higher SOA gain. This result aligns with established reservoir computing theory[9–11], which states that the total fading memory is limited by the number of independent internal variables (i.e., the number of virtual nodes) and by the length of the feedback loop. As nonlinearity increases, memory depth typically decreases, thereby defining the trade-off that determines the reservoir memory capacity[12].
This tunability highlights that our reservoir architecture allows for the precise control of the trade-off between fading memory and nonlinearity, thus enabling the system to be optimized for the specific requirements of different machine learning tasks.

The Echo State Property (ESP) ensures that the reservoir's internal state becomes asymptotically independent of its initial condition and is driven solely by the input signal. This property is essential for stable and reproducible learning in reservoir computing[2].
In conventional ESNs, ESP is often associated with a spectral radius below one. However, in physical systems like ours, which are governed by continuous feedback dynamics rather than discrete weight matrices, the ESP must be verified through direct observation of fading memory and input-driven convergence [2,9,13].
In our system, ESP is governed by the feedback strength, which is controlled by both VOA attenuation and nonlinear gain, as determined by the SOA voltage[1]. As shown in the simulations in Supplementary Fig. 2, appropriate tuning of these parameters ensures the decay of past input influence and maintains stable reservoir dynamics, while avoiding regions of oscillation or chaos, which are typically observed when the SOA is operated closer to or within the saturation regime in similar systems[14], thus being detrimental to the execution of machine learning tasks. To experimentally validate ESP-like behavior, we conducted a fading memory test using isolated picosecond input pulses. As shown in Supplementary Fig. 3, the reservoir response was recorded over multiple round trips via oscilloscope measurements (Supplementary Fig. 3(b)) and compared with the simulated behavior (Supplementary Fig. 3(c)). The progressive decay of the response in both cases confirms that the system forgets its initial state and reacts primarily to the current input, thus satisfying the expected conditions for ESP.

Supplementary Note 3. Single-bit versus dense encoding methods

A single-node, time-delayed RC approach relies on creating virtual nodes through time-division multiplexing by modulating the amplitude of a CW source, as detailed in the Methods section of the main manuscript entitled "*Information encoding and readout*". Shorter memory loops reduce latency but can impact performance accuracy by limiting the number of multiplexed virtual nodes required for accurate output classification.
Our approach addresses this limitation by applying parallel processing and a high-density encoding scheme. This generates pulse trains in which the amplitude carries the information to be processed, and the number of nodes determines the pulse duration. In our experiments, pulse widths ranged from 480 ps (with 1 bit per cavity round trip and 161 nodes) to 42 ps (with 488 bits per cavity round trip and 4 nodes per bit). The high-density encoding as was used for the nonlinear channel recovery task provides a significant increase in device throughput. Specifically, we encoded information at 6.5 Gbit/s per wavelength channel using the high-density encoding scheme, compared to 13.4 Mbit/s with the low-density encoding scheme.
The differences between these two encoding schemes are illustrated in Supplementary Fig. 4. 100 points of the MG time trace (a) were sampled and further encoded using both (b) low-density and (c) high-density schemes. The first approach requires 100 round-trips (each comprising 161 nodes) to input and process the sequence, whereas high-density encoding requires only 2 cavity round-trips (50 bits per round trip and 4 virtual nodes). This scheme, combined with wavelength-division multiplexing, further increases the

processing capacity and has been used in our telecom recovery task, for which Supplementary Fig. 5 shows representative bit sequences before and after processing.

Supplementary Note 4. Throughput scalability and energy efficiency of our device

Supplementary Note 4.1. Computational speed and maximum system throughput

We can calculate the device's processing speed in terms of multiply–accumulate operations per second (MAC/s) using the following equation, which was adapted for a reservoir following the approach of Nakajima et al.[15]:

$$S_{MAC} = \frac{n_c(MAC_p + MAC_{en})}{\tau_r} = \frac{n_c(N_v \cdot n_b \cdot C_f + n_b \cdot R_m \cdot C_f')}{\tau_r}, \quad (16)$$

where n_c is the number of wavelength channels and n_b is the number of bits or data points encoded per round-trip of duration τ_r (device latency, i.e., the time required to complete a single computational step). $\mathrm{MAC_p}$ and $\mathrm{MAC_{en}}$ are the multiply–accumulate contributions from processing and encoding, respectively. N_v is the number of virtual nodes (in our case, one). $C_\mathrm{f} = 6$ and $C_\mathrm{f}' = 3$ account for operations on complex numbers (node states, weights, and inputs), and $R_\mathrm{m} = 20$ is the number of random masking levels.

The number of tera-operations per second (TOPS), including the nonlinear operations arising from the activation function, can be calculated as:

$$S_{TOPS} = \frac{2 \cdot n_c(O_n \cdot MAC_p + MAC_{en})}{\tau_r}, \quad (17)$$

where $\mathrm{O_n}$ is the total number of reservoir operations per round-trip. In our device $O_\mathrm{n} = 10$, which accounts for the nonlinear computational steps[1], while the factor of 2 accounts for complex variable types.
Using the encoding rate per channel, $f_\mathrm{enc} = n_\mathrm{b}/\tau_\mathrm{r}$, speed can be written as:

$$S_{TOPS} = 2 \cdot n_c \cdot f_{enc} \cdot (O_n \cdot N_v \cdot C_f + R_m \cdot C_f'), \quad (18)$$

For the case of the nonlinear recovery task, when applying Eqs. (16) and (18), we obtain (respectively) $\mathrm{S_{MAC}} = 2.7\mathrm{MAC/s}$ and $\mathrm{S_{TOPS}} = 20$ TOPS.

In practice, throughput scalability is limited by two independent physical mechanisms. First, the SOA carrier recovery time (τ_rec) sets the maximum encoding rate as $1/\tau_\mathrm{rec}$, which corresponds to the smallest time delay of each virtual node, i.e., the masking rate (See the Methods section entitled "*Information encoding and readout*" in the main manuscript):

$$f_\mathrm{mask} = N_\mathrm{v} \cdot f_\mathrm{enc} \leq \frac{1}{\tau_{rec}}, \quad (19)$$

in which N_v is the number of virtual nodes. Operating beyond this rate results in dynamic gain compression and signal distortion, which hampers signal fidelity.
Furthermore, we impose a power-budget constraint on the device's circulating energy to prevent SOA gain saturation, requiring:

$$E_\mathrm{T} = \frac{P_\mathrm{T}}{f_\mathrm{mask}} = \frac{P_\mathrm{T}}{N_\mathrm{v} \cdot f_\mathrm{enc}} < E_\mathrm{sat}, \quad (20)$$

where E_T and P_T are the total circulating energy and power, respectively, while $\mathrm{E_{sat}}$ is the saturation energy.

[1] The contribution to the nonlinear computational steps arises from the application of the nonlinear operators acting on the electric field, as defined in Eqs. (10–12). Specifically, Eq. (10) involves two nonlinear operations, Eq. (11) adds two more, and Eq. (12) introduces one additional operation, for a total of five nonlinear operations. Because the electric field is complex, this number is then multiplied by a factor of 2, resulting in 10 nonlinear operations overall.

For indium phosphide SOAs, as used in our setup, the typical saturation energy is $E_{\mathrm{sat}} \approx 1$ pJ, and the carrier recovery time τ_{rec} falls within the hundreds of picoseconds range. We can estimate the circulating power from the average output power, which ranges from 100 μW to 300 μW per input channel. Considering the maximum value of 300 μW, assuming negligible losses, and scaling by up to five channels, Eq. (20) gives a maximum circulating energy of approximately 0.06 pJ. Therefore, Eq. (20) is satisfied for all tasks considered.

Finally, by substituting Eq. (19) into Eq. (18), the absolute upper throughput bound for distortion-free, high-speed operation is

$$S_{TOPS}^{max} = 2 \cdot \frac{N_{\mathrm{v}}}{\tau_{\mathrm{rec}}} \cdot \left(O_{\mathrm{n}} \cdot N_{\mathrm{v}} \cdot C_{\mathrm{f}} + R_{\mathrm{m}} \cdot C_{\mathrm{f}}'\right). \qquad (21)$$

This equation relates the maximum computational speed per channel to the number of virtual nodes and the dynamics of the SOA carriers.

By applying Eq. (21) to the channel recovery task, and considering the input power levels used, we obtain a maximum throughput of $\mathrm{S}_{\mathrm{TOPS}}^{\mathrm{max}} = 24$ TOPS per channel. This upper limit is six times higher than the demonstrated speed, as it is fundamentally limited by the electro-optical encoding equipment, which prevented the device from fully utilizing its potential. This limitation can be alleviated by increasing the number of channels, which aligns well with the methodology applied in our work.

The broadband response and low-loss operation of our device make it inherently compatible with dense wavelength division multiplexing (DWDM). In principle, standard DWDM grids could support up to approximately 80 channels over a single-mode fiber. To demonstrate this scalability, we experimentally generated 9 evenly spaced wavelength channels centered near 1550 nm, separated by about 0.15 nm (see Supplementary Fig. 6 (b)), using modulation instability from a single continuous-wave source. These channels were subsequently amplified with an erbium-doped fiber amplifier and spectrally flattened with a waveshaper. Due to the nature of their generation and the limited number of available equipment components (e.g., intensity modulators and arbitrary waveform generators), it is not possible to encode arbitrary data separately on each channel. We can however encode copies of the same input bit sequence for processing on each channel. Thus, we applied a four-level pulse-amplitude modulation (PAM-4) encoding scheme to inject and process 1200 bits for a sequence classification task. This approach highlights the scalability of our platform, where both dense temporal encoding and parallel wavelength multiplexing contribute independently to increased throughput without additional hardware complexity.

Supplementary Fig. 6 (a) shows the normalized mean square error and the bit error rate (BER) for the bits of each of the detected channels, with values below 10% for all nine channels. The variation in BER across channels is due to crosstalk arising from broad spectral filtering. The well-defined three-eye openings of the diagrams reported in Supplementary Fig. 6 (d), which were obtained by considering three of the nine channels (Ch1, Ch5, Ch9), demonstrate the high accuracy of our device in retrieving the initial bit sequence, as shown in Supplementary Fig. 6 (c).

Supplementary Note 4.2. Power consumption of our device

Using Eq. (20), we can estimate the power consumption of the device's processing unit[16,17], which includes the entire figure-eight cavity configuration and all its components, such as the SOA, the SW, and the VOA (see Supplementary Fig. 1) as

$$P_{\mathrm{PU}}^{\mathrm{W}} \sim \left[N_{\mathrm{m}} \cdot \left(E_{\mathrm{m}} \cdot B_{\mathrm{r}} + P_{\mathrm{m}}\right) + P_{\mathrm{VOA}} + P_{\mathrm{SOA}}\right] \cdot N_{\mathrm{p}} \qquad (22)$$

For the channel recovery task, where $N_{\mathrm{m}} = 1$ is the total number of IMs employed for the reservoir virtual nodes, $E_{\mathrm{m}} = 0.2$ pJ/b is the energy consumption of the modulator, B_{r} is the processing speed expressed in bits per second, $P_{\mathrm{m}} = 61$ mW is the power required to drive the modulator for input masking, and $P_{\mathrm{VOA}} =$

25 mW is the maximal power consumption associated with the VOA in the operating conditions of our experiments. P_{SOA} = 0.5 mW is the SOA power consumption, while N_{p} = 1 is the number of physical nodes in the reservoir. By replacing these values in Eq. 20, we obtain $P_{\mathrm{PU}}^{\mathrm{W}}$~87mW. For nonlinear channel recovery, considering a total throughput of 20 TOPS, the energy consumption results as approximately 4.4 fJ OP^{-1}.

Peripheral components used for data encoding, readout, and acquisition operate alongside the processing unit but do not contribute directly to computation. Therefore, following the methodology adopted in previous studies[18], we exclude their power consumption from the evaluation of our device's computational efficiency. Moreover, these components can be replaced with more energy-efficient hardware in future implementations. For completeness, however, we still report their power usage in our current setup. The multi-output CW laser used to generate the input channels consumes 18 W. The IMs, one for the processing unit (masking) and five for channel encoding, consume approximately 400 mW overall. The AWGs, comprising 2 channels each (i.e., with three AWGs employed, we have five channels for data encoding, and one for masking), draw approximately 150 W in total. Photodetectors and other encoding and readout components contribute negligibly to power consumption, typically below the milliwatt range.

Importantly, our architecture uses dense encoding, where multiple bits are injected during each cavity round-trip per channel. This enables the throughput needed to scale up without requiring additional AWGs, IMs, or lasers. The total aggregate rate can be expressed as $R_{\mathrm{total}} = n_{\mathrm{c}} \cdot R_{\mathrm{sym}} \cdot b$, with $\mathrm{R_{sym}}$ the symbol rate per channel, and b the number of bits per symbol. Currently, we use binary modulation (b = 1), so each symbol carries 1 bit. However, higher-order modulation offers a straightforward way to lower peripheral demands while boosting throughput. For example, adopting 64-QAM (b = 6) would replace the current five data channels with a single channel at the same symbol rate. Under this setup, AWG consumption would drop from approximately 150 W to about 25 W, IM usage from around 0.4 W to 70 mW, and the number of active laser channels from five to one, resulting in a 40–55% reduction in laser wall-plug power, depending on the overhead. Additional improvements can be achieved by switching from discrete CW sources to an integrated multi-wavelength laser array. This eliminates idle losses per channel, saving several watts while maintaining a high optical signal-to-noise ratio.

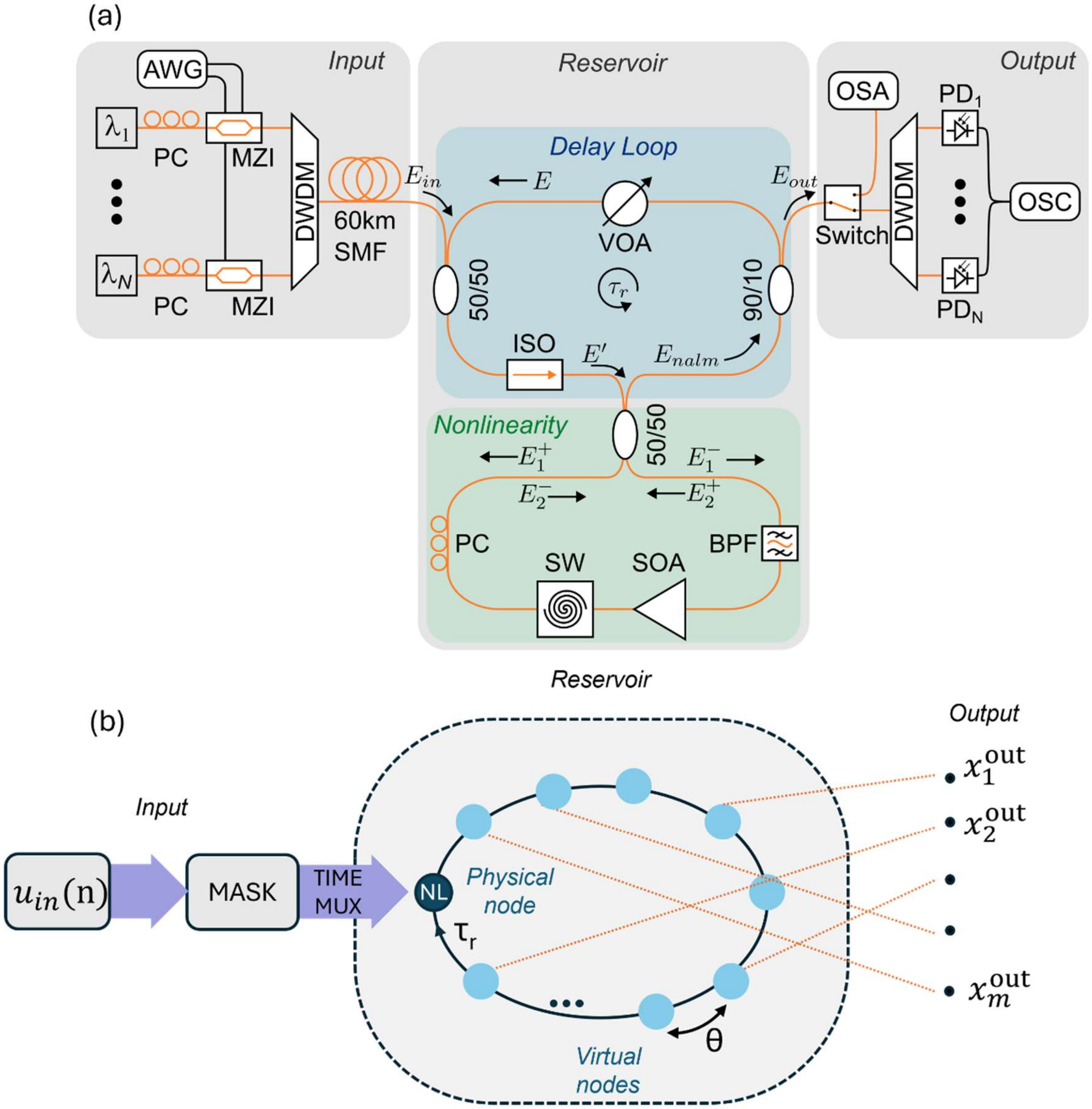

Supplementary Fig. 1. Device architecture and working principle. (**a**) The input field $\boldsymbol{E}_{\mathbf{in}}$ carries data encoded through amplitude modulation of the continuous-wave (CW) source, achieved using an arbitrary waveform generator (AWG) and a Mach-Zehnder intensity modulator (IM). The field $\boldsymbol{E}'$ is the result of the combination of the externally injected field $\boldsymbol{E}_{\mathbf{in}}$ and the delayed propagating field, $\boldsymbol{E} \equiv \boldsymbol{E}(\boldsymbol{t} - \boldsymbol{\tau_r})$, originating from previous round-trips. $\boldsymbol{E}_{\mathbf{1}}^{\pm}$, propagating in opposite directions, originate from the field $\boldsymbol{E}'$, which splits in the 50:50 coupler. $\boldsymbol{E}_{\mathbf{2}}^{\pm}$, which are the fields after nonlinear interaction and amplification, recombine again in the coupler, producing the NALM output field, $\boldsymbol{E}_{\mathbf{NALM}}$. $\boldsymbol{E}_{\mathbf{out}}$, which is the field for collected output. (**b**) Schematic representation of the architecture implemented by our device in (**a**), a delay-based reservoir computing system using a single nonlinear node and virtual nodes generated via time multiplexing. The input sequence $\boldsymbol{x}_{\mathbf{in}}$ is first multiplied by a random mask and then time-multiplexed to modulate a continuous signal. This signal is injected into a delay loop containing a single physical nonlinear node (NL) with a feedback delay $\boldsymbol{\tau}$, forming a dynamical system. The loop is sampled at intervals of $\boldsymbol{\theta}$, generating a set of virtual nodes that emulate a high-dimensional recurrent network. The output is obtained through a linear combination of the reservoir states.

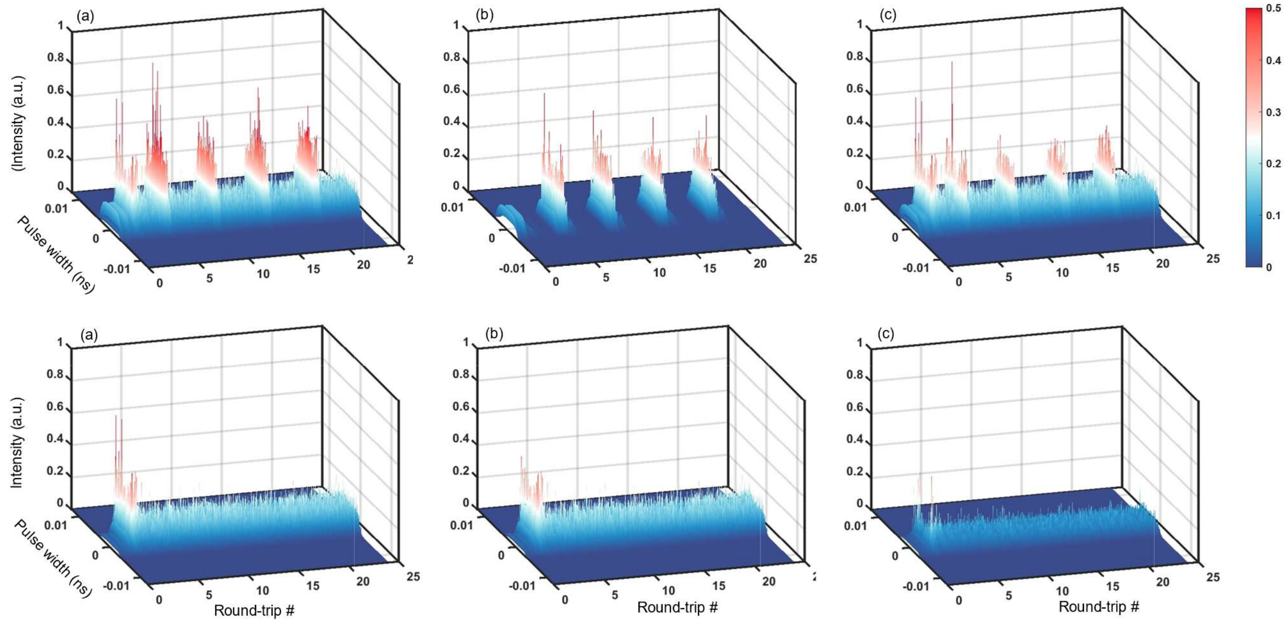

Supplementary Fig. 2. Simulated pulse propagation, showing the presence of a fading memory response. The intensity and pulse width of the overall field E are plotted over multiple round-trips by varying the SOA gain $\boldsymbol{g}$, as well as the VOA attenuation. The gain values are $\boldsymbol{g} = \mathbf{6.4\ dB}$ and $\boldsymbol{g} = \mathbf{10.3\ dB}$ (indicated on the top (a-c) and bottom (d-f) graphs), while the VOA attenuation is increased from $\mathbf{A_{VOA}} \approx \mathbf{0\ dB}$ in (a) and (d) to $\mathbf{A_{VOA}} \approx \mathbf{1\ dB}$ in (b) and (e), reaching $\mathbf{A_{VOA}} \approx \mathbf{5.2\ dB}$ in (c) and (f). These results illustrate the effect of the reservoir parameters on the system's memory and stability. Balancing the trade-off between attenuation and gain ensures the exponential decay of the internal state influence, consistent with ESP, as observed in (c), (e), and (f), while excessive gain leads to pulsing or chaotic regimes, as seen in (a), (b), and (d).

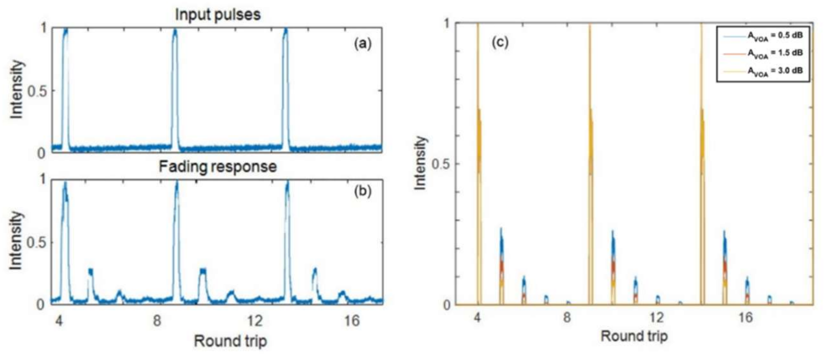

Supplementary Fig. 3. Experimental and theoretical verification of fading response. Isolated input pulses (**a**) show a decaying response over round trips (**b**), thus confirming ESP-like dynamics in the operational regime. (**c**) Simulation case study demonstrating that ESP-like behavior is maintained across the tested parameter space for various VOA attenuation values.

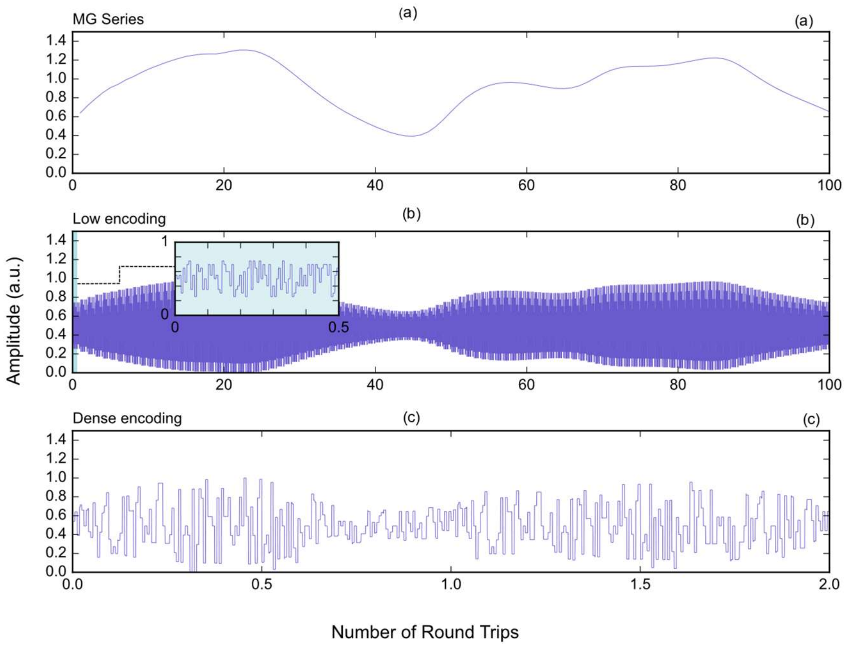

Supplementary Fig. 4. Comparison between low-and high-density encoding schemes. RC encoding of a 100-point MG signal (**a**) can be achieved using two different methods: low-density (**b**) and high-density (**c**) encoding. In the low-density case (**b**), one MG data point is encoded per round trip. Conversely, in the high-density case (**c**), 100 MG data points are encoded per round trip. In both scenarios, the number of virtual nodes, N_{v}, is 161, and a random mask with 20 levels is applied.

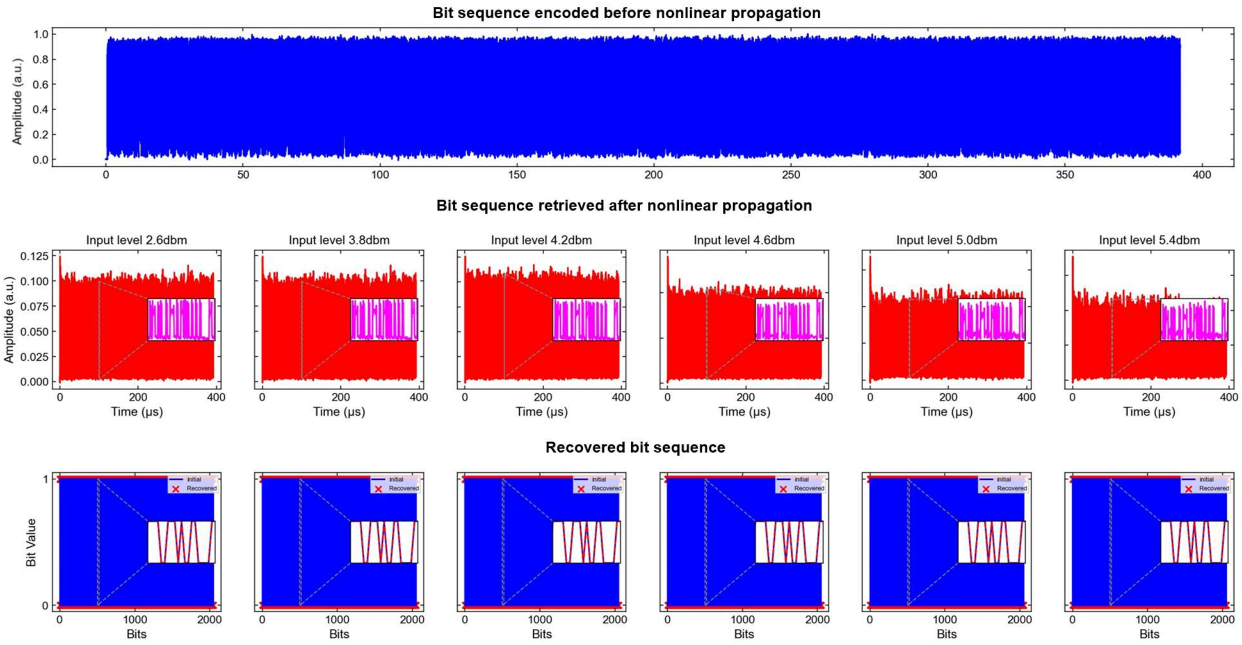

Supplementary Fig. 5. Experimental bit sequences before and after nonlinear propagation, and recovery by the photonic reservoir computer. (Top) Original bit sequence (blue) encoded onto the amplitude of a continuous-wave laser before propagation. (Middle row) Measured amplitude traces (red) after propagation through 60 km of standard single-mode fiber at different input power levels (2.6–5.4 dBm), showing distortion caused by chromatic dispersion and nonlinear effects, including cross-phase modulation and four-wave mixing. The overlaid magenta trace in each inset shows a zoomed-in portion of the corresponding distorted sequence for clarity. (Bottom row) Example of recovered bit sequences from one of the five employed channels (specifically, channel λ_4), after processing by the photonic reservoir computer. The blue trace represents the original (input) bit

sequence, while the red "X" markers indicate the bits recovered by the system. The high overlap between the two confirms successful retrieval despite severe waveform degradation.

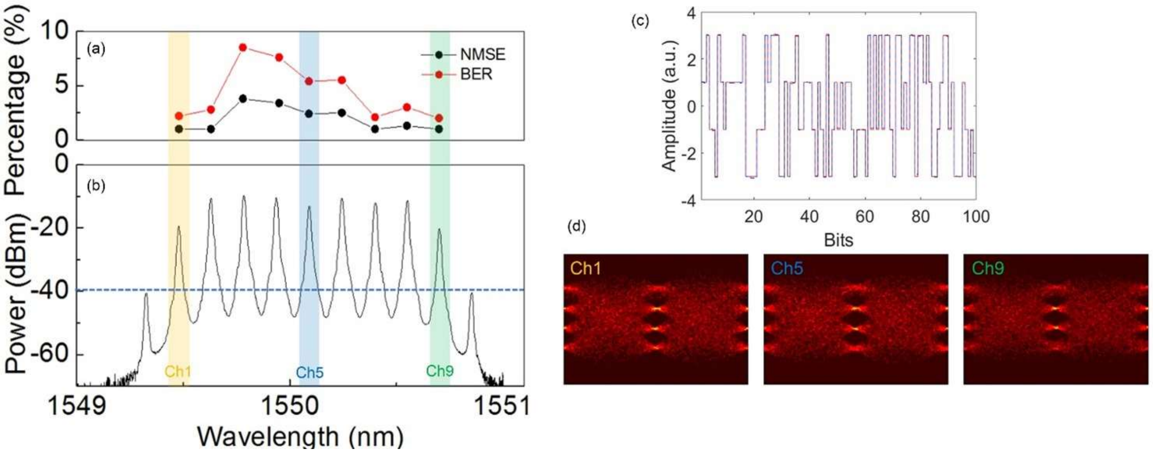

Supplementary Fig. 6. PAM4 signal recovery using nine wavelength channels. The BER and NRMSE values are reported in (**a**) for each of the nine channels represented in (**b**). (**c**) shows the recovered PAM-4 sequence after ML training. (**d**) displays the eye diagrams of the reconstructed signals on (Ch1, Ch5, Ch9). The shaded regions in orange, blue, and green highlight the BER values in (**a**) for the channels Ch1, Ch5, and Ch9, respectively.

Supplementary Table 1. Tunable and fixed setup parameters used in the simulations.

Parameter	Symbol	Value
Group velocity dispersion (fiber) @1550 nm	β_2^{fb}	-70 ps^2 km^{-1}
Group velocity dispersion (SW) @1550 nm	β_{2sw}	-8 ps^2 km^{-1}
Third-order dispersion (SW) @1550 nm	β_{3sw}	-0.3 ps^2 km^{-1}
Loss coefficient (fiber)	α_{fb}	0.2 dB km^{-1}
Loss coefficient (SW)	α_{SW}	-3 dB cm^{-1}
Kerr nonlinear coefficient (SW)	γ	200 W^{-1} m^{-1}
Input coupling ratio	k_{in}	0.5
NALM splitter/coupling ratio	k	0.5
VOA coupling ratio	k_{VOA}	0 - 1
Output coupling ratio	k_{out}	0.9
SOA driving voltage	V_{SOA}	0.2 V – 1.6 V
SOA gain	g	0.5 dB - 10.4 dB
VOA driving voltage	V_{VOA}	~0.5 V – 3.5V
VOA attenuation	A_{VOA}	~0 dB - 9.2 dB

Supplementary References

1. Appeltant, L. *et al.* Information processing using a single dynamical node as complex system. *Nat. Commun.* **2**, 468 (2011).
2. Yildiz, I. B., Jaeger, H. & Kiebel, S. J. Re-visiting the echo state property. *Neural Netw.* **35**, 1–9 (2012).
3. Köster, F., Yanchuk, S. & Lüdge, K. Master Memory Function for Delay-Based Reservoir Computers With Single-Variable Dynamics," in IEEE Trans. Neural Netw. Learn. Syst. **35**, 7712-7725 (2024)
4. Stelzer, F., Röhm, A., Lüdge, K. & Yanchuk, S. Performance boost of time-delay reservoir computing by non-resonant clock cycle. *Neural Netw.* **124**, 158–169 (2020).
5. Appeltant, L. Reservoir computing based on delay-dynamical systems. *PhD thesis, Vrije Universiteit Brussel and Universitat de les Illes Balears (2012).*
6. Fan, C., Li, L. & Yu, F. Soliton solution, breather solution and rational wave solution for a generalized nonlinear Schrödinger equation with Darboux transformation. *Sci. Rep.* **13**, 9406 (2023).
7. Govind Agrawal. *Nonlinear Fiber Optics*. *Academic Press* (2013).
8. Connelly, M. J. Wideband semiconductor optical amplifier steady-state numerical model. *IEEE J. Quantum Electron.* **37**, 439–447 (2001).
9. Manjunath, G. & Jaeger, H. Echo State Property Linked to an Input: Exploring a Fundamental Characteristic of Recurrent Neural Networks. *Neural Comput.* **25**, 671–696 (2013).
10. Jaeger, H. Long Short-Term Memory in Echo State Networks: Details of a Simulation Study. Technical Report 27, *Jacobs University Bremen*, Germany (2012).
11. Boikov, I. K., Brunner, D. & De Rossi, A. Evanescent coupling of nonlinear integrated cavities for all-optical reservoir computing. *New J. Phys.* **25**, 093056 (2023).
12. Dambre, J., Verstraeten, D., Schrauwen, B. & Massar, S. Information processing capacity of dynamical systems. *Sci. Rep.* **2**, 514 (2012).
13. Frank, S. L. & Čerňanský, M. Generalization and Systematicity in Echo State Networks. *Proceedings of the Annual Meeting of the Cognitive Science Society* **30**, 865–870 (2008).
14. Aadhi, A. *et al.* Highly reconfigurable hybrid laser based on an integrated nonlinear waveguide. *Opt. Express* **27**, 25251 (2019).
15. Nakajima, M., Tanaka, K. & Hashimoto, T. Scalable reservoir computing on coherent linear photonic processor. *Commun. Phys.* **4**, 20 (2021).
16. Kikuchi, K. Fundamentals of coherent optical fiber communications. *J. Lightw. Technol.* **34**, 157–179 (2016)."
17. Wang, B., de Lima, T. F., Shastri, B. J., Prucnal, P. R. & Huang, C. Multi-Wavelength Photonic Neuromorphic Computing for Intra and Inter-Channel Distortion Compensations in WDM Optical Communication Systems. *IEEE J. Sel. Top. Quantum Electron.* **29**, 1–12 (2023).
18. Gu, Z., Ma, Q., Gao, X., You, J. W. & Cui, T. J. Direct electromagnetic information processing with planar diffractive neural network. *Sci. Adv.* **10**, eado3937 (2024).
