# Supplementary material for: Scalable photonic reservoir computing for parallel machine learning tasks
Source: Nat Commun. 2025 Dec 31;17:1225. doi: 10.1038/s41467-025-67983-z (PMC12864733; doi:10.1038/s41467-025-67983-z)
Supplement: Supplementary file 2 — Transparent Peer Review file [file 41467_2025_67983_MOESM2_ESM.pdf]

nature portfolio

Peer Review File

Scalable Photonic Reservoir Computing for Parallel Machine Learning Tasks

Corresponding Author: Dr Luigi Di Lauro

This file contains all reviewer reports in order by version, followed by all author rebuttals in order by version.

Version 0:

Reviewer comments:

Reviewer #1

(Remarks to the Author)
The manuscript introduces a photonic neural network architecture built using 4×4 multimode interferometers (MMIs) and a proposed multi-operand microring resonator (MMORR) for nonlinear operations. The primary goal is to provide a scalable and energy-efficient solution for deep learning acceleration by addressing limitations of Mach-Zehnder interferometer (MZI) and microring resonator (MRR) based networks. The authors present theoretical models, perform FDTD simulations, and evaluate the architecture on standard image classification datasets (MNIST, FMNIST, CIFAR-10). The work claims high compute density and low power consumption. The proposal of MMIs combined with nonlinear MMORRs represents a potentially promising approach to optical computing and deep learning acceleration. The authors attempt to incorporate nonlinear optical activation functions directly into the architecture.

However there are multiple critical weaknesses. It is not clear how the operation of the MMORR-based nonlinear unit is implemented and the simulations fail to illustrate the computing mechanism convincingly. How is the nonlinearity generated in the MMORRs? Why is this approach is superior or even viable compared to known methods. The experimental validations are insufficient. In particular the experimental results on CIFAR-10 show only a 54.5% accuracy—significantly below state-of-the-art—and the authors do not explain this shortcoming or compare fairly with digital or hybrid solutions. The compute density calculation ignores critical physical constraints like metal routing and electronic interface area, significantly inflating the reported performance. No evidence is provided for multi-mode utilization, even though final metrics are derived based on it.

The paper claims power efficiency but does not rigorously detail the power consumption of each optical or electrical component, nor does it benchmark against known designs or standards.

The paper does not discuss practical issues such as variability, fabrication tolerance, and bandwidth associated with optical nonlinearity.

Reviewer #2

(Remarks to the Author)
In the manuscript “Scalable Photonic Reservoir for Parallel Machine Learning Applications”, the authors design a figure-eight shaped nonlinear amplifying loop mirror architecture for photonic reservoir computing, claiming that a 20-TOPS-level of computing power and a femto-joules-per-operation-level of efficiency are achieved. Even though the reported performances and capability seem to be superior, several questions need to be clarified to make the statements convincing.

1. In figure 1, the authors proposed a double ring-resonator structure to implement an all-optical reservoir computing system. While the physical architecture is clearly depicted, the manuscript lacks detail regarding how the weights associated with the SOA and VOA are trained or simulated.

2. The manuscript presents several results on machine learning tasks. However, the specific neural network architectures employed to achieve these results are not described in sufficient detail. What is the exact configuration of the network that is

deployed and mapped onto the proposed photonic hardware? Providing a clear and comprehensive description of the network structure is essential for ensuring the clarity of the presented results.

3. The customized ring-resonating structure has a high risk for undesired self-exciting oscillations. How will you eliminate such noises to make the computing stable and the results repeatable?

4. In Figure 4, the authors presented the original undistorted telecom signal and the distorted signal after propagation. However, such a figure only visualizes a common nonideal propagation phenomenon, and has little relation with the proposed research. The authors may need to provide the recovered signal shape after the proposed photonic reservoir computing.

5. Figure 4 presents only a basic illustration of the time traces of the modulated field intensity before and after propagation. However, the manuscript lacks a detailed analysis or interpretation of these results, which may leave readers confused about the significance and implications of the figure. More detailed numerical analysis for the performance is necessary to make the research more convincing.

6. The authors mentioned “scalable” in the title of the manuscript, however, there seems to be no discussions of computing scalability in the results section. More extra details, comparisons, figures, curves, and / or discussions that related to the network scale and system scalability is necessary to support the claims in the title.

7. In the system throughput analysis section, the manuscript does not clarify the clock frequency assumed in the numerical calculations. Since throughput is inherently tied to the system’s operating frequency, the lack of this information hinders a proper assessment of the reported performance.

8. In the energy efficiency analysis section, it remains unclear whether the authors have accounted for the energy consumption and potential speed limitations introduced by peripheral components. These include, for example, the data feeding mechanisms, data acquisition systems, and the voltage drivers required for modulating the weights. The authors are encouraged to clarify whether these peripheral factors were considered in the analysis, and if not, to discuss their possible influence on the practical implementation of the proposed system.

9. While the manuscript introduces a new NALM-based architecture, it lacks a comprehensive comparison with other existing photonic computing approaches in terms of key performance metrics such as throughput, energy efficiency, and computational accuracy. Including such comparisons would greatly strengthen the manuscript by highlighting the relative advantages of the proposed design.

10. In addition, it would be beneficial if the authors could provide baseline performance metrics for the demonstrated computing tasks using conventional methods (e.g., electronic computing method). Presenting such benchmarks would offer valuable context for evaluating the practical benefits of the proposed photonic implementation.

In conclusion, the submitted manuscript reported a novel photonic reservoir computing architecture with claimed promising performance and capability, but these mentioned questions need to be settled to further make the manuscript more comprehensive and convincing.

Reviewer #3

(Remarks to the Author)
This paper proposes and implements a wavelength-division-multiplexed (WDM) reservoir computing (RC) system that enables parallel neuromorphic computation. As WDM-based RC has already been experimentally demonstrated in previous studies (e.g., [1]), the main contribution of this paper lies in its implementation of a nonlinear delay system called the nonlinear amplifying loop mirror (NALM). Although this system utilizes gain saturation in a semiconductor optical amplifier (SOA), the principle itself has already been reported in prior works [2,3], and thus lacks sufficient novelty. While the experimental demonstration is intriguing, the reported computational performance (20 tera operations per second) is lower than that of previous studies ([1] 800 TOPS, [4] 200 TOPS), and therefore does not offer the level of impact typically expected for a publication in Nature Communications. Accordingly, I do not recommend publication in Nature Communications. I believe this work would be more suitably presented in journals such as Scientific Reports or Communications Physics, once the following concerns are addressed.
Major comments:
- The introduction should briefly review prior works on WDM-based approaches (e.g., [1,4,5]) and clearly delineate the differences between those studies and the present work. The authors should also cite earlier approaches that utilize SOA gain saturation (e.g., [2,3,6]) and clarify how their approach differs from them. In particular, [3,6] introduce nonlinearity via cross-gain saturation by injecting light into both ports of the SOA, and since the conceptual similarities are evident, the distinctions must be made clear.
- The relationship between the mathematical model in the Supplementary Information, and the experimental parameters (e.g., Gs and T_VOA shown in Fig. 2) is unclear. The correspondence between physical quantities such as Gs and T_VOA and the parameters in equations (S1–S15) should be explicitly stated.
- Additionally, in Fig. 2, the units of T_VOA and Gs are given in volts (V), which are device-dependent parameters; as a result, readers cannot determine what is actually being optimized. It would be preferable to express these quantities in terms of optical attenuation or gain, rather than voltage.
- In reservoir computing, it is generally expected that the feedback gain (or spectral radius of a connection matrix within a

reservoir) should be set to satisfy the echo state property (ESP) [7]. Can the authors discuss how to select the physical parameter to satisfy the ESP for the proposed configuration? In particular, it seems plausible that chaotic transitions could occur in this system if we set the strong feedback gain of the SOA. I guess that such region should be avoided.
- Lastly, in the nonlinear equalization experiments, it was stated that four virtual nodes were used. In such a case, the delay length would be significantly longer than a single symbol period (i.e., the inverse of the baud rate). Then, assuming the simplified reservoir dynamics described by $x(n) = f(Wx(n-p) + Mu(n))$, the parameter p would be considerably larger than 1. Why does the system still perform well under this condition? I apologize if I have misunderstood something, but clarification would be appreciated.

[1] https://www.nature.com/articles/s42005-021-00519-1
[2] https://opg.optica.org/oe/fulltext.cfm?uri=oe-20-20-22783&id=242389
[3] https://opg.optica.org/oe/fulltext.cfm?uri=oe-30-13-22871&id=476798
[4] https://www.nature.com/articles/s41467-024-55172-3
[5] https://opg.optica.org/optica/fulltext.cfm?uri=optica-10-11-1478&id=541462
[6] https://ieeexplore.ieee.org/abstract/document/10368205
[7] https://www.sciencedirect.com/science/article/abs/pii/S0893608012001852

Version 1:

Reviewer comments:

Reviewer #2

(Remarks to the Author)
The revision offers more details regarding figure-eight shaped nonlinear amplifying loop mirror architecture for photonic reservoir computing and has addressed some of my concerns. There still remain some issues that should be addressed to support this paper's claims.
1.In the revised manuscript, the authors have clarified the factors contributing to the system's scalability, such as encoding speed and the number of wavelength channels. However, it remains unclear whether the gains in computational throughput from these factors are fundamentally bounded. I recommend adding relevant analyses to help readers understand the potential upper limits of the system's capability. Furthermore, while the authors state that the improvement in computational throughput requires no additional computational units, it would be valuable to discuss whether there are other hardware costs involved-for example, increased demands on lasers and AWGs, and the associated higher power consumption.
2.The revised manuscript compares the proposed system with DSP only in terms of latency and energy consumption, without detailing the performance of the two hardware platforms on the same tasks, e.g., NRMSE in the time-series prediction task and BER in the nonlinear telecom signal recovery task. Since latency and energy consumption comparisons are only meaningful under comparable task performance, adding this information would better demonstrate the advantages of the proposed method.
3.In the nonlinear telecom signal recovery task, the manuscript states that the proposed hardware can recover distorted signals and effectively reduce BER. However, the presented results lack a baseline comparison, making it difficult to assess whether the method's performance is truly competitive. Including relevant baselines would help readers better appreciate the method's effectiveness.
4.In Table 1, the authors compare their work with existing photonic computing approaches, but omit prior implementations of photonic reservoirs, which are highly relevant to the proposed system. Including comparisons with such works is necessary to provide a more comprehensive evaluation.
In conclusion, the current version still contains several issues and a second-round revision that addresses these major concerns is recommended.

Reviewer #3

(Remarks to the Author)
I appreciate the authors' thorough and thoughtful responses to our previous comments. The revised manuscript addresses all of my concerns. I recommend the acceptance of the manuscript.

Version 2:

Reviewer comments:

Reviewer #2

(Remarks to the Author)
I appreciate the authors' thorough and constructive responses to the reviewers' comments. The revised manuscript satisfactorily addresses all previous concerns, and I recommend it for publication in Nature Communications.

Open Access This Peer Review File is licensed under a Creative Commons Attribution 4.0 International License, which permits use, sharing, adaptation, distribution and reproduction in any medium or format, as long as you give appropriate credit to the original author(s) and the source, provide a link to the Creative Commons license, and indicate if changes were made.
In cases where reviewers are anonymous, credit should be given to 'Anonymous Referee' and the source.
The images or other third party material in this Peer Review File are included in the article's Creative Commons license, unless indicated otherwise in a credit line to the material. If material is not included in the article's Creative Commons license and your intended use is not permitted by statutory regulation or exceeds the permitted use, you will need to obtain permission directly from the copyright holder.
To view a copy of this license, visit https://creativecommons.org/licenses/by/4.0/

Reviewer #1 (Remarks to the Author):

We sincerely thank the reviewer for taking the time to assess our Manuscript. However, we respectfully note that several of the comments raised appear to pertain to a different topic or paper and do not reflect at all he content, methodology, or scope of our work.
To clarify, our Manuscript focuses on a photonic reservoir computing architecture based on a nonlinear amplifying loop mirror (NALM), with emphasis on dense temporal encoding, wavelength-multiplexed multi-task processing, and experimental demonstrations across several machine learning benchmarks, including XOR tasks, time-series predictions, and nonlinear channel recovery. In contrast, the reviewer's comments appear to refer to the use of multimode interferometers and multi-operand microring resonators, which are *not* employed in this Manuscript, for image classification on datasets such as MNIST and CIFAR-10, which *are unrelated* to the methodology and scope of our work.

Reviewer #2 (Remarks to the Author):

In the manuscript "Scalable Photonic Reservoir for Parallel Machine Learning Applications", the authors design a figure-eight shaped nonlinear amplifying loop mirror architecture for photonic reservoir computing, claiming that a 20-TOPS-level of computing power and a femto-joules-per-operation-level of efficiency are achieved. Even though the reported performances and capability seem to be superior, several questions need to be clarified to make the statements convincing.

We thank the reviewer for considering our work and for recognizing the promising performance and potential of our approach. To enhance the clarity and overall quality of the Manuscript, we have provided a detailed, point-by-point response to all the concerns raised.

1. In figure 1, the authors proposed a double ring-resonator structure to implement an all-optical reservoir computing system. While the physical architecture is clearly depicted, the manuscript lacks detail regarding how the weights associated with the SOA and VOA are trained or simulated.

Response

Our approach is based on time-delay reservoir computing, as opposed to a spatial reservoir with trainable internal weights. In this architecture, computation is distributed across virtual nodes, which are time-multiplexed samples of a single nonlinear dynamical system governed by a delayed feedback loop.
Importantly, the internal reservoir weights are not trained. Instead, they are fixed and randomly defined through the input masking procedure, as previously established (Appeltant et al., 2011). In our system, the random mask is implemented via a sample-and-hold mechanism, and the values of the mask define the reservoir weights.
This was discussed in Section S1 of the Supplementary Information in the earlier submission on line 32 (now lines 32-33).

The virtual nodes represent transient states of the nonlinear system, sampled at equally spaced time intervals over the delay period.
The only trainable weights in the reservoir are the readout weights, for which a linear regression is performed on the collected node states to produce the output (see Methods). Indeed, the reservoir approach eliminates the need for internal weight optimization, which is a core advantage of this paradigm.
To further clarify: our system does not train internal weights via the SOA or VOA. Rather, there are fundamental factors that shape the reservoir's dynamic response, which we have demonstrated through a detailed theoretical analysis using our model. Using our model, we explicitly show how modifying these physical parameters affects key reservoir properties, such as memory and nonlinear transformation capabilities, as was detailed in the Supplementary Information (lines 115–120 in the earlier submission).

Moreover, we would like to point out that our device doesn't implement a ring-resonator structure, but rather a figure-8 shaped cavity, which is fundamentally different in terms of field propagation and nonlinear interaction.

ACTION TAKEN

- Besides having been mathematically derived on lines 114–129 of the Supplementary Information, we have also revised lines 32–33 of the Supplementary Information and lines 103–107 of the Manuscript. In the latter, we now state: '*Consistent with the RC paradigm, training was applied exclusively to the output weights, while the internal node connection weights remained fixed. These internal weights are inherently defined by the device configuration and the specific components employed, such as the SOA, the integrated SW, and the variable optical attenuator (VOA) (see also Supplementary Information).*' This clarifies that only the output weights are trained in the reservoir computing framework and further explains the roles of the SOA and VOA in the reservoir dynamics.

2. The manuscript presents several results on machine learning tasks. However, the specific neural network architectures employed to achieve these results are not described in sufficient detail. What is the exact configuration of the network that is deployed and mapped onto the proposed photonic hardware? Providing a clear and comprehensive description of the network structure is essential for ensuring the clarity of the presented results.

Response

It is indeed true that we didn't sketch the corresponding network architecture. However, we consistently stated that our NALM configuration implements a reservoir computer (e.g., lines 58-68 in the earlier Manuscript version, now lines 73-96), which is a recurrent network architecture requiring the presence of a feedback loop in addition to an overall, tunable nonlinearity that our system can produce. Details on how the network configuration, i.e., the reservoir, is established on the device setup were presented in the Methods section (see paragraph beginning on line 345, under 'Information encoding and readout', and paragraph beginning on line 319 under 'Device Architecture', respectively, in the earlier Manuscript version).

ACTION TAKEN

- We have clarified the analogy between the RC structure and our NALM configuration, as outlined on lines 103-107.
- We have plotted the reservoir structure in the new Figure S.1, added to the Supplementary Information, where Eqs. S1-S3 describe its behavior.

3. The customized ring-resonating structure poses a high risk of undesired self-excited oscillations. How will you eliminate such noises to make the computing stable and the results repeatable?

Response

We thank the reviewer for bringing this important point to our attention. Self-excited oscillations are a known dynamic in ring resonator-based structures; however, our architecture, based on a figure-eight configuration rather than a resonant cavity, with an integrated spiral waveguide as the nonlinear element, inherently mitigates this issue. This is in line with the findings in our previous work (Aadhi et al., 2019). We maintain the SOA bias below the onset of nonlinear gain regimes, where Ikeda-type instabilities emerge. By operating safely within this threshold, the system remains in a stable regime. As was detailed in the Methods section (lines 280-281 in the earlier Manuscript version, now on lines 332-334), we operate the SOA below saturation, far from the region in which gain instabilities and spontaneous oscillations typically emerge. Furthermore, our implementation of the NALM architecture includes a narrow (~200 GHz) bandpass filter (see Fig. 1). This element limits the spectral bandwidth and effectively suppresses chaotic oscillatory behavior, such as square-wave and fast oscillations. In addition, this element limits modulation instability gain, therefore, ensuring the stability of computing results.

Experimentally, we identify in Fig. 2(a–c) the specific regions of parameter space, defined by the SOA bias voltage and VOA attenuation, where the device achieves high accuracy and low NRMSE. These regions are characterized by stable operating regimes that are free from oscillatory noise, which was validated across benchmark tasks. Finally, the experimental results presented in Figs. 3 and 5, demonstrated across multiple independent wavelength channels and diverse benchmark tasks, proving the high repeatability and computational stability of our system. The consistent performance across XOR, Mackey-Glass, NARMA10, and nonlinear channel recovery tasks further reinforces the robustness of our architecture against noise-induced instabilities.
Therefore, we confirm that we have not observed any self-excited oscillations that would introduce parasitic effects in our system.

ACTION TAKEN

- We have revised lines 332-334 of the Manuscript to clarify this matter further. We now state: '*The SOA operates between the linear and nonlinear regimes, far from saturation, to avoid instabilities. These include self-oscillation and chaotic behaviors, as well as noise amplification and signal fluctuations, which would be detrimental to the current tasks.*'

4. In Figure 4, the authors presented the original undistorted telecom signal and the distorted signal after propagation. However, such a figure only visualizes a common non-ideal propagation phenomenon and has little relation to the proposed research. The authors may need to provide the recovered signal shape after the proposed photonic reservoir computing.

5. Figure 4 presents only a basic illustration of the time traces of the modulated field intensity before and after propagation. However, the manuscript lacks a detailed analysis or interpretation of these results, which may leave readers confused about the significance and implications of the figure. More detailed numerical analysis for the performance is necessary to make the research more convincing.

Response

We want to clarify that the distorted signal shown in Figure 4(b) is not a generic or idealized example of signal degradation, but the result of actual experimental measurements acquired after propagation through a 60 km standard single-mode fiber link, which introduces both dispersion and nonlinear impairments, including cross-phase modulation and four-wave mixing (as was described on lines 180–188 in the earlier Manuscript version, now on lines 213-221). Such a distorted telecom signal serves as the actual input to our device, which recovers the original signal upon machine learning training.

While the system's performance is already quantified in Figure 5 via the bit error rate (BER), as a standard metric in telecom applications, we agree that showing qualitative examples of the recovered waveforms would strengthen the Manuscript.

We want to clarify the following points regarding the analysis and interpretation of these results: The performance of our device in recovering these distorted signals is quantitatively presented in Figure 5(a), where we report the BER as a function of input power across five wavelength channels. This BER analysis compares the signal quality before and after processing. It is discussed in detail in the Manuscript on lines 222–223, where we state: '*The bit error rate (BER, the metric that quantifies the percentage of misclassified bits in the recovered sequence).*' Fig. 5(b) further emphasizes this comparison by plotting both the pre-processed (green markers) and post-processed (purple markers) BER, clearly showing the benefit of using the device.

This experimental analysis is supported by numerical simulations, performed using our custom model based on the generalized nonlinear Schrödinger equation, as described in the *Methods* section and derived in Supplementary Information (Section S.1). The simulation results, shown as black dots in Figure 5(a), closely match the experimental BER trends, thus validating the model and our overall approach.

The signal to be recovered was a digital sequence of bits (0 and 1) encoded onto the modulated amplitude of a continuous-wave laser. As a result, the recovered signal consists of discrete digital levels, not a continuous waveform. Thus, the concept of a 'shape' is not well-defined in this context, as it applies specifically to analog signals.

ACTION TAKEN (in response to points 4 and 5)

- Accordingly, we have added new plots in Fig. 5, showing in (c) the original bit sequence alongside its distorted carrier signal after nonlinear propagation through the 60 km fiber at 5.2 power; in (d) the corresponding recovered sequence after processing by our device, as also detailed on lines 241-245. We have also added an Extended Data figure, which shows all traces before and after recovery, for each power level used.

6. The authors mentioned “scalable” in the title of the manuscript, however, there seems to be no discussions of computing scalability in the results section. More extra details, comparisons, figures, curves, and / or discussions that related to the network scale and system scalability is necessary to support the claims in the title.

Response

We thank the reviewers for bringing this to our attention.
We demonstrate the scalability of our network in terms of computational throughput (TOPs/s), as defined in Eq. (5) of the Manuscript. This throughput scales directly with both the encoding speed and the number of wavelength channels. Unlike traditional time-multiplexed optical RC devices, where encoding speed is fundamentally limited by the cavity round-trip time, our dense encoding technique significantly increases the information throughput per temporal frame (quantified by the parameter n_b in Eq. (5), enabling higher data rates without requiring additional computational units.

This scalability is further enhanced through multi-level encoding, as illustrated in Section S.4 of the Supplementary Information, where we employ a PAM-4 scheme. Additionally, we demonstrate the potential for broadband frequency conversion using four-wave mixing, which could scale system performance to the peta-operations-per-second range (see lines 310-314 of the Manuscript). In parallel, scalability is also achieved by increasing the number of wavelength channels and enabling multi-processing. This was experimentally validated using nine parallel wavelength channels for simultaneous data encoding, as described in the same section.

ACTION TAKEN

- We have clarified, throughout the Manuscript, that scalability refers specifically to computational throughput. To further emphasize this, we have added the following sentence on lines 202-203 of the Manuscript: ‘*We demonstrate system scalability through both increased bit density and parallel channel encoding, enabling scaling of computational throughput without requiring additional physical nodes.’*

- We have also revised section S.4 of the Supplementary Information on lines 213-216: ‘*This approach highlights the scalability of our platform, where both dense temporal encoding and parallel wavelength multiplexing contribute independently to increased processing throughput without additional hardware complexity.*’

- We have revised the Abstract to better reflect the key novelties, including the scalable aspect.

7. In the system throughput analysis section, the manuscript does not clarify the clock frequency assumed in the numerical calculations. Since throughput is inherently tied to the system’s operating frequency, the lack of this information hinders a proper assessment of the reported performance.

Response

In the domain of reservoir computing, the fundamental clock frequency is commonly associated with the inverse of

the round-trip time of the feedback loop, i.e., the inverse of the system latency (Hülser et al., 2022). In other papers, however, the clock frequency is defined in relation to the desynchronization factor, also referred to as the asynchronous clock frequency (Tang et al., 2023). In this context, what we refer to as latency has also been referred to as the round-trip time, defining the clock cycle.

Although the clock frequency was not explicitly defined in the earlier version of the Manuscript, we did discuss the system throughput in relation to latency, which was quantified as 75 ns, on lines 242–246 in the earlier Manuscript version, now on lines 289-291. Furthermore, in Eqs. (6) and (7), the throughput is accounted for in terms of system speed and supports the performance claims of our device.

ACTION TAKEN

- To ensure clarity and remove any ambiguity, we have reorganized the text on lines 289-296 and explicitly mentioned the fundamental clock frequency of our reservoir, clarifying its physical origin. We also discuss its relation to system latency and how it defines the reservoir's throughput and, consequently, the overall device performance. We report an extract of the edited text here: '*The reservoir's throughput is directly linked to its fundamental clock frequency, defined as the inverse of the round-trip time. In our system, this corresponds to 13.3 MHz* (75 ns round-trip)*, setting the device's intrinsic operational speed and hence its inherent throughput...*'

8. In the energy efficiency analysis section, it remains unclear whether the authors have accounted for the energy consumption and potential speed limitations introduced by peripheral components. These include, for example, the data feeding mechanisms, data acquisition systems, and the voltage drivers required for modulating the weights. The authors are encouraged to clarify whether these peripheral factors were considered in the analysis, and if not, to discuss their possible influence on the practical implementation of the proposed system.

Response

We appreciate the reviewer's insightful comment. We estimated the energy efficiency of our system using commonly adopted methods reported in previous studies, which we cite in our Manuscript. In some of these studies, for example, in Gu et al (Gu et al., 2024), the energy analysis primarily considers the consumption of the processing unit itself, treating peripheral components (e.g., data encoding and readout equipment) separately. This distinction is justified, as peripheral equipment can often be replaced with more energy-efficient alternatives in future implementations.

In our case, we report the energy consumption of the processing unit of the device, which includes the entire figure-of-eight cavity configuration and all its components (see Fig. 1). Since our reservoir is predominantly composed of integrated and passive elements, the main energy contribution comes from these components, with low power drawn by the integrated active elements, such as the SOA used in the processing unit.
We acknowledge that the power consumption of the VOA was not included in our previous calculation. However, we confirm that the energy consumption associated with the processing unit remains of the same order of magnitude as previously calculated and reported in our work.

ACTION TAKEN

- We have recalculated the power consumption of the processing unit in Eq. 7, adding the VOA power draw on lines 474-475, where we state: '$P_{VOA} = 25\ mW$ *is the maximal power consumption associated with the VOA in the operating conditions of our experiments.*'

- In response to the reviewer's recommendation, on lines 478-487 in the Methods section, we now provide a clear distinction between the energy consumption of (i) the processing unit alone and (ii) the processing unit including peripheral components. Here, we state that '*Peripheral components used for data encoding, readout,*

and acquisition operate alongside the processing unit but do not contribute directly to computation. Therefore, following the methodology adopted in previous studies 60, we exclude their power consumption from the evaluation of the device's computational efficiency. Moreover, these components can be replaced with more energy-efficient hardware in future implementations. For completeness, however, we report their power usage in our current setup. The multi-output CW laser used to generate the input channels consumes a total of 18 W. The IMs, one for the processing unit (masking) and five for channel encoding, consume approximately 400 mW overall. The AWGs, comprising 2 channels each (three AWGs employed, five channels for data encoding, and one for masking), draw approximately 150 W in total. Photodetectors and other encoding and readout components contribute negligibly to power consumption, typically at a level below the milliwatt range.'

- We corrected the value of energy efficiency reported in the Manuscript, which is now estimated at 4.4 fJ/OP.
- We have also specified throughout the Manuscript that we refer to energy efficiency related to the processing unit exclusively, including adding a note in Table 1.

9. While the manuscript introduces a new NALM-based architecture, it lacks a comprehensive comparison with other existing photonic computing approaches in terms of key performance metrics such as throughput, energy efficiency, and computational accuracy. Including such comparisons would greatly strengthen the manuscript by highlighting the relative advantages of the proposed design.

Response

We thank the reviewer for this valuable suggestion.
While the current version of the Manuscript already introduces performance metrics that allow for a quantitative evaluation of our device in terms of computational throughput, energy efficiency, and accuracy, we agree with the reviewer that further details are beneficial.

Consistent with our previous replies, Eqs. (5–7) define the throughput and energy efficiency metrics used to assess our system. Additionally, Table 1 in the Manuscript provides a comparative overview of key performance indicators, including the computational throughput (as defined by the maximum number of MAC operations per second, as seen in (Bernstein et al., 2023)), energy efficiency, and the type of machine learning task, for a range of state-of-the-art photonic computing platforms.

Regarding accuracy, this is quantified using the normalized root mean square error (NRMSE), as introduced in Eq. (4) and explicitly referenced on lines 108-109 of the Manuscript, or in terms of BER, as reported on lines 222-225 of the Manuscript.

ACTION TAKEN

- To further strengthen this comparison as requested, we have added a new column to Table 1 that summarizes the reported computational accuracy for each listed architecture per specified ML task.

10. In addition, it would be beneficial if the authors could provide baseline performance metrics for the demonstrated computing tasks using conventional methods (e.g., electronic computing method). Presenting such benchmarks would offer valuable context for evaluating the practical benefits of the proposed photonic implementation.

Response

We appreciate the suggestion and recommendation, which enable us to further expand on the benefits of our proposed implementation. The latency and power efficiency of DSPs are highly dependent on the design of the

ASICs that support the algorithm. Nonetheless, the energy consumption of electronics for multiplication and additions can be found to be 208.5 fJ/bit and 26 fJ/bit, respectively, in (Pillai et al., 2014), and a standard Feed-Forward Equalizer algorithm for an NRZ 25 GBaud requires 33 operations (17 multiplications, 16 additions). Thus, a DSP alternative to our method would require ~ 3 pJ/bit/ per channel. The latency of the DSP algorithm is often highly dependent on the ASIC architecture, but conventionally ranges from 100 ns to the ms timescale.

ACTION TAKEN

- For comparison, we have included, on lines 293-304, the general performance of DSP algorithms and the range of performances observed in published research on optical signal processing in peer-reviewed journals. In particular, we state: '*As quantitatively derived in Eqs. (6) and (7), the device achieves a maximum throughput of 20 TOPS while maintaining a BER as low as 2×10−4 for telecom signal recovery, outperforming traditional digital signal processing-based equalization methods (DSP), which are less effective at mitigating nonlinear distortions over long distances and consume significantly more power. In this regard, our architecture offers greater energy efficiency per operation than other photonic counterparts, whose processing unit consumes only 4.4 fJ/OP. This is because the device's reservoir consists of integrated and passive elements; therefore, the main energy consumption arises from these components, with negligible power drawn by the active elements, such as the SOA and IM. For comparison, DSP algorithms average energy consumption of approximately three pJ/bit per channel for our communication protocol, with latency between 100 ns and 1 us depending on the system clock speed64, while reported optical signal processing methods' performances range from 20 to 200 ps latency and 0.1 to 1 pJ/bit channel.*'

- We have included two new articles as references for this comparison: (Liu et al., 2024) (112 Gbps/lane, 0.1 pJ/bit, ~ ps range) and (Staffoli et al., 2024) (20 Gbps/lane, latency ~ 200 ps, 1.25 pJ/bit). We used these as the performance range anticipated for the current telecom signal processing implementations, as stated on lines 300-304.

In conclusion, the submitted manuscript reported a novel photonic reservoir computing architecture with claimed promising performance and capability, but these mentioned questions need to be settled to make further the manuscript more comprehensive and convincing.

We sincerely thank the reviewer again for the very constructive and insightful feedback, as well as for recognizing the potential of our work. We have carefully considered all the suggestions and believe that we have addressed them thoroughly in the revised Manuscript.

Reviewer #3 (Remarks to the Author):

This paper proposes and implements a wavelength-division-multiplexed (WDM) reservoir computing (RC) system that enables parallel neuromorphic computation. As WDM-based RC has already been experimentally demonstrated in previous studies (e.g., [1]), the main contribution of this paper lies in its implementation of a nonlinear delay system called the nonlinear amplifying loop mirror (NALM).

We thank the reviewer for bringing this aspect to our attention. We were indeed aware of [1] work that had already cited it in our Manuscript. However, we would like to take this opportunity to clarify further the key differences between our work and references [1], [4], and [5], particularly regarding WDM-related aspects, which were also raised in a later comment (Reviewer Comment #1).

A major distinction between our system and that of [1,4,5] lies in the scalability of the architecture, in terms of both reservoir structure and detection overhead, which directly impacts processing capability, adaptability, and overall hardware efficiency.
While these prior works differ in implementation, they share several common limitations:

1. The systems lack tunability, with the activation function, fading memory inherently constrained by the design, limiting flexibility and adaptability to tackle different machine learning tasks.

2. The reservoir dynamics remain linear, with nonlinearity applied only at the input/output stages via electronics, introducing Optical-to-Electrical-to-Optical (OEO) latency.

3. These systems do not support true task-level parallelism (or concurrency, defined in the Manuscript), as multiple wavelength or spatial channels are typically used to decompose and distribute a single task across parallel streams, rather than enabling independent execution of multiple distinct tasks.

In [1], the system employs spatio-temporal multiplexing, where each bit is delayed using fixed delay lines and processed in separate reservoir units that act as masking nodes. This enables interleaved processing (i.e., not real concurrency), hence adding throughput latency in input/readout. Moreover, operation is constrained to specific input laser conditions (e.g., $\theta = T/N$), and each reservoir unit requires a separate pulsed laser source, further limiting flexibility and scalability. The system also requires 32 photodiodes per channel, resulting in significant detection overhead.

In [4], a similar *interleaving* strategy replaces recurrent feedback with a linear structure where delayed bit streams emulate memory. However, identical to [1], this fixes the node dimensionality and limits scalability. While frequency channels can parallelize a task by mimicking the identical input (as we also demonstrate in Figure 3 in the Manuscript), this system does not support task differentiation across channels. Importantly, the use of interleaving for parallelization compromises true concurrency, as it introduces preprocessing latency that ultimately limits the computational throughput, the actual processing speed of the device.

In [5], multiple wavelength channels are only used to increase reservoir complexity by mixing frequency-domain nodes through a cascaded architecture. However, the system does not support independent reservoir tasks. Instead, it increases the effective dimensionality of a single task through spectral coupling, without enabling per-channel flexibility or parallel execution of tasks with varying complexity.

Across all three systems, nonlinearity is confined to the input and output layers and implemented electronically, making the reservoir dynamics effectively linear. This reliance on electronic activation introduces OEO latency and limits the exploitation of ultrafast, nonlinear effects intrinsic to the optical approach.

In contrast, besides computational scalability, our system is designed to allow a tunable control of the reservoir properties. We use CW lasers with amplitude modulation to encode information, and reservoir properties are reconfigurable via the configuration tunable parameters, such as the SAO and VAO driving voltages. This enables us to process data with varying levels of complexity across multiple wavelength channels, all within a single shared reservoir. Our architecture supports any signal speed, limited only by the upper limit of input/readout electronics bandwidth, enabling a universal, fully reconfigurable optical computing platform. This flexibility is demonstrated through our applications in XOR logic, time-series classification, and nonlinear channel equalization, all of which utilize a unified reservoir.

Regarding the reviewer's comment: '*the main contribution of this paper lies in its implementation of a nonlinear delay system called the nonlinear amplifying loop mirror (NALM).*'

Our choice to employ a NALM configuration to realize our processing device is guided by its proven efficacy in generating highly stable and controllable nonlinear interactions, essential for optical reservoir computing (Kues et al., 2017; Aadhi et al., 2019; Xiong et al., 2023). Previous approaches, such as those based on unidirectional cavities or cross-gain modulation, have limitations including fixed nonlinear dynamics and reduced task versatility (Duport et al., 2012, 2016; Vandoorne et al., 2008).

In contrast, the NALM architecture inherently supports robust and controllable nonlinear response, relying promising results demonstrated our previous studies (Aadhi et al. 2019, Optics Express, 27, 25251-25259), where the figure-eight cavity configuration demonstrated high tunability and a very rich nonlinear dynamics response, beneficial for advanced optical computing and reservoir computing tasks, in terms of fine classification of inputs. Additionally, earlier studies, such as those by Adams et al. (1994) and Kues et al. (2017), underline the NALM's intrinsic advantages in stable nonlinear dynamics, making it particularly attractive for reliable optical reservoir computing applications.

Besides the novel configuration we implemented, our work introduces several key architectural and operational novelties not only in terms of speed. This may have been misinterpreted in the earlier Manuscript version due to a lack of clarity. We want to summarize the novelty aspects of our work here as follows.

i. True concurrent multi-task processing, with each wavelength channel independently masked and processed in parallel within the same reservoir units (stated on lines 83–90, demonstrated in the parallel execution of the MG time-series and NARMA10 prediction tasks, along with the multi-channel recovery task).
ii. Computational throughput scalability is achieved by combining dense temporal encoding (each channel carries multiple bits per round-trip, thereby enhancing information density and computational speed) with parallel wavelength channels, allowing each channel to carry multiple bits per round-trip. This significantly enhances information density and computational efficiency without requiring additional components or modifications to the device architecture (see lines 208-212).
iii. Tunable nonlinearity embedded directly in the reservoir, enabled by the NALM mechanism and the presence of an SOA operating in an unsaturated regime, allowing precise control over the nonlinear response and avoiding the limitations associated with cross-gain saturation.
iv. High energy-efficiency compared to previous demonstrations, even those chip-based (see Table 1 in the Manuscript).

Taken together, our work presents a qualitatively different approach to optical neuromorphic computing, focusing on scalable, reconfigurable, and energy-efficient parallel processing, with experimentally demonstrated performance from our device.

ACTION TAKEN

- To address the reviewer's comments regarding other WDM-based approaches, we have substantially revised the Manuscript introduction (lines 50-64) to discuss better the differences of our approach with respect to earlier works. We now state: '*Recent efforts to increase computational throughput in reservoir computing have focused on faster electro-optical modulation and advanced data encoding schemes, such as pulse-amplitude modulation*[27–29] *(PAM), often paired with wavelength-division multiplexing (WDM). However, these methods frequently rely on sequential sweeping across WDM channels*[30–32]*, or time-interleaving techniques*[33–37]*, which both introduce throughput latency and prevent the concurrent execution of independent tasks. Further attempts to scale performance further, by adding components or increasing the number and dimensionality of computational layers and readout components*[5,37]*. Typically, this results in decreased energy efficiency and processing speeds.*
  Moreover, existing WDM-based RC architectures[36–38] *exhibit limited tunability. For instance, both the reservoir memory and activation function are inherently fixed by design constraints. At the same time, nonlinearity is introduced electronically at the input/output stage, resulting in optical-to-electrical-to-optical (OEO) latency and limiting the richness of reservoir dynamics. Such constraints significantly reduce system flexibility and adaptability for diverse ML tasks. Additionally, architectures employing spectral mixing approaches*[38] *encounter nonlinear crosstalk*[39]*, restricting the number of independently encoded channels and consequently degrading system accuracy and scalability.'*

- We have revised the abstract to clearly present the key novelties upfront, ensuring a prompt understanding of the main contributions of the work.

- We have further clarified the motivation and advantages that led us to exploit the NALM configuration for reservoir computing (lines 67-71). We state '*We leverage the NALM configuration for its proven ability to*

generate highly stable and nonlinear dynamics[40–43]*, essential for optical reservoir computing. These dynamics enhance the reservoir's computational capacity by enabling complex input mapping into a high-dimensional state space, thereby improving sensitivity to extremely small signal variations*[44,45]*. The intrinsic stability of the NALM ensures consistent classification performance, even under noisy or fluctuating input conditions.*'

-Although this system utilizes gain saturation in a semiconductor optical amplifier (SOA), the principle itself has already been reported in prior works [2,3], and thus lacks sufficient novelty.

Response

We thank the reviewer for their valuable and insightful comments, which allow us to clarify in more detail why our device avoids SOA gain saturation and how this significantly enhances our system's tunability and control, thus underpinning the novelty and impact of our work.

We fully agree with the reviewer that SOAs are already widely used for processing units in optical reservoir computing. Indeed, seminal works by Vandoorne et al. (2008) and Duport et al. (2012, 2016) operate their reservoir devices in a unidirectional configuration with SOAs in the saturation gain regime, resulting in a fixed nonlinear response resembling a tanh function. Similarly, Refs. [2,6] showcase cross-gain modulation (XGM) approaches, where nonlinear processing explicitly relies on operation within the saturation region, as also emphasized in Ref. [3]. In contrast, our approach leverages fundamentally different dynamics by intentionally avoiding SOA gain saturation. As mentioned earlier and noted in the earlier Manuscript version as well (lines 280-281), our device does not operate in the gain saturation regime: "*The SOA is operated between the linear and nonlinear regimes, far from saturation*." Therefore, we believe that these conceptual and operational differences position the novelty of our work relative to prior studies, thereby reinforcing its significance.

Specifically, gain saturation restricts the dynamic range of the nonlinear response, as the SOA gain plateaus at saturation, substantially limiting the ability to finely tune the reservoir nonlinearity and thus its optimal configuration for ML tasks. By operating the SOA primarily below the saturation regime, we can precisely control the gain via driving voltage adjustments, enhancing the tunability and performance of our device.

Quantitatively, this tunability is clearly demonstrated in our parameter maps (Fig. 2), where the joint optimization of the SOA gain and VOA settings leads to high accuracy in machine learning tasks. Specifically, we used the n-bit delayed XOR task, which requires precise control over both the memory and nonlinearity of the reservoir, to benchmark this capability. We achieved 100% classification accuracy for the 2-bit XOR task, 97% for the 3-bit XOR task, and 80% for the 4-bit XOR task. Notably, such task-specific optimizations would not be possible if the SOA gain was fixed at saturation levels.

Additionally, operating the SOA away from saturation significantly enhances system stability by mitigating intensity fluctuations and self-oscillation effects commonly observed in saturated gain regimes, particularly in NALM configurations(Aadhi et al., 2019). As previously noted in our response to Reviewer #2's question, these instabilities are detrimental to the reservoir's ability to classify input signals reliably(Appeltant et al., 2011), thereby impairing overall computational performance.

ACTION TAKEN

- To address comments regarding the operational regimes of the SOA used in this work, and to emphasize the key differences between our approach and earlier methods based on saturated gain operation, we have clarified the text by adding the following text on lines 73–79 '*Our device features an integrated nonlinear spiral waveguide (SW) and a semiconductor optical amplifier (SOA), which both modulate the NALM overall nonlinear transfer function (i.e., the reservoir activation function). In contrast to previous RC implementations that rely on SOAs operating in saturation, whether in unidirectional configurations*[46–48] *or through cross-gain modulation*[49,50]*, we set the SOA below saturation for all our experiments. Saturated SOAs exhibit plateaued gain, limiting the*

dynamic range and tunability of the nonlinear response. The unsaturated regime, instead, allows precise tunability of the gain, and therefore of the reservoir activation function.'

- We provided further explanation on lines 154-162 regarding the nonlinearity arising from our device, which is distinguished from previous gain-saturation reservoir computing-based devices reported. Here we state '*During such an interaction, an increase in the amplification gain causes the SOA to contribute additional nonlinear response, thereby enhancing classification performance when further nonlinear contributions are required*[53] *without altering the physical reservoir topology. This unsaturated operational regime enables us to implement a controllable mechanism mediated by tunable parameters, allowing for the exploitation of nonlinearities arising from controlled optical feedback and intensity-dependent interference within the NALM. This approach allows for precise and reconfigurable reservoir functionality. In contrast to the cross-gain saturation-based nonlinearities demonstrated in earlier works* [49,50]*. Our architecture provides greater flexibility and control over reservoir dynamics.*'

-While the experimental demonstration is intriguing, the reported computational performance (20 tera operations per second) is lower than that of previous studies ([1] 800 TOPS, [4] 200 TOPS), and therefore does not offer the level of impact typically expected for a publication in Nature Communications. Accordingly, I do not recommend publication in Nature Communications. I believe this work would be more suitably presented in journals such as Scientific Reports or Communications Physics, once the following concerns are addressed.

Response

We respectfully disagree with the reviewer's comment and appreciate the opportunity to clarify both the computational performance in relation to the studies cited ([1], [4]) and the novelty of our work.
Regarding the processing speed in [1], a careful review shows that the experimentally demonstrated throughput is explicitly stated as approximately 20 to 44 TMAC/s per wavelength channel, not 800 TOPS. The mentioned "petascale" performance in that work is a theoretical extrapolation, assuming extremely dense WDM encoding that was not experimentally realized.

Regarding [4], it reports a processing speed of 200 TOPS, which is achieved under a feed-forward architecture with multi-channel sequential injection. As we have also discussed in reply to point #1, this method introduces preprocessing latency and does not support the concurrent execution of distinct tasks. Their throughput metric reflects an aggregate of serialized operations, not a truly parallel processing capability. As a result, the claimed figure does not represent real-time, task-level concurrency. Indeed, when we decouple the N=8 delayed input copies, we obtain a speed of 200/8= 25 TOPS.

In contrast, our architecture achieved true concurrent parallel processing within a single reservoir unit. As emphasized in the Manuscript, our system supports the simultaneous execution of multiple, independently encoded WDM channels, eliminating the latency associated with sequential injection and enabling real-time, multi-task computation. Our reported performance of 20 TOPS, measured under this configuration, reflects a real, measured throughput in a parallel execution regime, rather than an extrapolated or serialized estimate, at only femtojoule-level energy per operation, with high accuracy for classification and regression tasks. These figures are based on performance metrics, which can be rigorously estimated using Eqs. (4-7).

We apologize if this aspect was not better emphasized in the earlier version of the Manuscript. We believe that these contributions, which directly address all the reviewers' criticisms and clarify aspects that may have been previously misunderstood or insufficiently discussed in our Manuscript, may help reassess the novelty and significance of our work.

Major comments:

1. The introduction should briefly review prior works on WDM-based approaches (e.g., [1,4,5]) and clearly delineate the differences between those studies and the present work.

Response

We have addressed this point in relation to the reviewer's initial remarks.

2. The authors should also cite earlier approaches that utilize SOA gain saturation (e.g., [2,3,6]) and clarify how their approach differs from them. In particular, [3,6] introduce nonlinearity via cross-gain saturation by injecting light into both ports of the SOA, and since the conceptual similarities are evident, the distinctions must be made clear.

Response

We have addressed this point in relation to the reviewer's initial remarks.

3. The relationship between the mathematical model in the Supplementary Information and the experimental parameters (e.g., Gs and T_VOA shown in Fig. 2) is unclear. The correspondence between physical quantities such as Gs and T_VOA and the parameters in equations (S1–S15) should be explicitly stated.

4. Additionally, in Fig. 2, the units of T_VOA and Gs are given in volts (V), which are device-dependent parameters; as a result, readers cannot determine what is actually being optimized. It would be preferable to express these quantities in terms of optical attenuation or gain, rather than voltage.

Response

We thank the reviewer for pointing out that these relationships were not reported in the Supplementary Information. We take this opportunity to provide additional details regarding the role of these parameters in the derived model. Our model, as defined in Eq. (S4), is based on a generalized nonlinear Schrödinger equation that describes the propagation of the optical field within the device. This equation includes a gain function, denoted G in Eq. (S4), with units of inverse length. The function G depends on the longitudinal coordinate z and the SOA driving voltage, which is reported in Fig. 2 and labeled as G_S.

G is numerically estimated by integrating the model developed by (Connelly, 2001), which accurately captures the internal physical processes of an InP–InGaAsP SOA, such as the one used in our experiments. This model accounts for recombination dynamics, spontaneous and stimulated emission, spatial dependence, and spectral response. Experimentally, the driving voltage can be mapped to amplification gain values (denoted, for instance, as g) and reported in decibels, provided the input power is known, using either manufacturer-supplied calibration curves or direct experimental characterization. If the SOA operates in the saturation regime, one can approximate $G \approx g/L$, where L is the length of the SOA active region. As our experiments are performed below the saturation regime, the gain function G is estimated using the model in (Connelly, 2001), based on the applied SOA driving voltage (i.e., G_S).
Accordingly, the driving voltage is used as an input parameter in our MATLAB-based numerical solver for Eq. (S4) and directly corresponds to the values reported in the Manuscript under the label G_S.
We accounted for the effect of the VOA on the optical field through the parameter T_{VOA}, which represents its driving voltage. In our model, this effect is incorporated via the dimensionless parameter k_{VOA}, defined in Eq. (S13) as a function of T_{VOA} and ranging from 0 (full attenuation, or 0% transmission) to 1 (no attenuation, or 100% transmission), and depending linearly on the attenuation values. As with the SOA, the VOA driving voltage can be related to specific attenuation values, expressed in dB, and either provided by the manufacturer or determined through experimental characterization.

ACTION TAKEN

- Following the reviewer's suggestion, we have now made explicit the correspondence between G_s, T_{VOA}, and the parameters used in equations (S1–S15) in the Supplementary Information, on lines 59-69 and lines 114-116, respectively, as well as on lines 124-125 in the Manuscript.

- We have updated the Supplementary Information to clearly distinguish between the gain function G used in the model and the experimental amplification gain, now introduced as g, expressed in decibels (dB) on lines 65-67.

- We have clarified on lines 114-116 of the Supplementary Information how the VOA, controlled by its driving voltage parameter, is linked to the model via a dimensionless scaling factor k_{VOA}. Percentage-based values of T_{VOA} have now been converted to decibels to maintain consistency throughout the Manuscript.

- To avoid confusion among parameters and the common physical quantities they refer to, we have relabeled the parameter T_{VOA} as V_{VOA}, the parameter G_s as V_{SOA}, introduced the SOA amplification gain g, and the attenuation levels A_{VOA} in all instances, in both the Manuscript and Supplementary Information.

- We have updated Fig. 2 to report attenuation and amplification gain values (now indicated as g vs A_{VOA}) in decibels. Along with their dB values, we still provide the respective driving voltage values (hence, V_{SOA} and V_{VOA})

- We have updated Table S1 to include the missing units for all parameters.

5. In reservoir computing, it is generally expected that the feedback gain (or spectral radius of a connection matrix within a reservoir) should be set to satisfy the echo state property (ESP) [7]. Can the authors discuss how to select the physical parameter to satisfy the ESP for the proposed configuration? In particular, it seems plausible that chaotic transitions could occur in this system if we set the strong feedback gain of the SOA. I guess that such region should be avoided.

Response

We thank the reviewer for this insightful comment. The ESP is indeed a key requirement in reservoir computing, as it ensures that the internal dynamics of the reservoir become asymptotically independent of initial conditions and are uniquely driven by the input signal. In other words, ESP ensures that past influences gradually vanish (i.e., the system "forgets" its initial conditions) and that the reservoir primarily reflects the current input. This property is fundamental to the execution of machine learning tasks in reservoir computing.
While it is commonly assumed that setting the spectral radius of the reservoir weight matrix below one ensures ESP, as pointed out in [7], this condition is neither necessary nor sufficient in general. The presence of ESP also depends on several additional factors, including the characteristics of the input signal (e.g., amplitude, frequency content, and dynamical richness), the type and strength of nonlinearity in the reservoir, and the input scaling. Nevertheless, the ESP can still hold when the spectral radius is greater than one [7] (Frank and Čerňanský, 2008; Manjunath and Jaeger, 2013)

<u>a) On selecting physical parameters to ensure ESP:</u>
In time-delay-based reservoir architectures like ours, where recurrence is physically implemented through a closed-loop fiber system with a single nonlinear node, the effective "internal connection strength", which would define the spectral radius in conventional networks, governs fading memory and stability, and is controlled by the feedback strength and the nonlinear gain (Appeltant et al., 2011). In our system, this is modulated through two key parameters: the VOA attenuation parameter A_{VOA} (controlled by V_{SOA}), which scales the intensity of the circulating signal after each round trip via k_{VOA} (see Eq. S13), and the SOA amplification gain g (controlled by V_{SOA}), which controls the gain and nonlinearity of the system (Eq. S4).

As shown in our simulations (Fig. S2), adjusting the VOA attenuation and SOA gain provides fine control over how much of the signal from previous round trips influences the current state. A sufficiently attenuated feedback loop ensures the exponential decay of memory traces, thereby satisfying ESP, whereas excessive retention can lead to instabilities or pulsing/chaotic regimes (see Fig. S2).

To experimentally verify this, we performed fading memory measurements (Fig. S3 (a)), in which isolated input pulses were injected into the loop and the resulting output was monitored over time. These tests confirmed that the influence of the input decays over successive round trips, as expected for ESP-compliant dynamics. In addition, our parameter sweep (see Table S1) confirms that ESP-like behavior is maintained across the operational range used in this work, as illustrated by the simulation case study shown in Fig. S3(b).

b) On the potential for chaotic dynamics:
We agree with the reviewer that operating the SOA at high gain may induce nonlinear effects such as self-pulsing or chaotic behavior. To avoid this, all experiments were conducted with the SOA operated well below saturation, using driving voltages in the range of 0.2–1.6 V, corresponding to moderate gain values of 0.5–10.4 dB. These values were selected based on simulation results and validated experimentally by monitoring fading memory, as described above.

This choice ensures that the feedback loop does not enter self-sustained oscillatory or chaotic regimes, both of which would violate the ESP. As further discussed in Section S.2 as well as in the Manuscript, increasing the SOA gain while keeping the feedback constant results in reduced fading memory, due to the intrinsic trade-off between nonlinearity and memory capacity in reservoir systems (Dambre et al., 2012).
We have previously studied such chaotic regimes in related systems, such as NALM laser configurations, where we observed that once chaos sets in, the system becomes insensitive to external input and thus unusable for reservoir computing tasks. Accordingly, we avoid these regimes to ensure robust, input-driven transient dynamics suitable for machine learning.

ACTION TAKEN

- We revised the title of Section S.2 of the Supplementary Information to "Reservoir Tunability and Echo State Property Verification". We added a new subsection (S.2.1) to define ESP in the context of physical reservoir computing. This includes a discussion of how ESP depends not only on spectral radius but also on input characteristics, nonlinearity, and feedback strength. We clarified that, in our delay-based photonic system, ESP is governed by VOA attenuation and SOA gain and is directly linked to fading memory and system stability.

- Captions of Figs. S2 and S3 have been updated to highlight how tuning feedback and gain controls fading dynamics and ESP.

6. Lastly, in the nonlinear equalization experiments, it was stated that four virtual nodes were used. In such a case, the delay length would be significantly longer than a single symbol period (i.e., the inverse of the baud rate). Then, assuming the simplified reservoir dynamics described by $x(n) = f(Wx(n–p) + Mu(n))$, the parameter p would be considerably larger than 1. Why does the system still perform well under this condition? I apologize if I have misunderstood something, but clarification would be appreciated.

Response

This is a very insightful question, and we thank the reviewer for raising it. We agree that in conventional reservoir computing schemes, where only a single bit is injected per round-trip, a delay significantly longer than the symbol period results in a large parameter p, which can degrade performance.

In our implementation, we introduce a dense bit encoding scheme, where up to 488 bits are injected per round-trip. Each bit is encoded over approximately 42 ps using time-multiplexing via a sample-and-hold procedure, as described in the Methods section ('Information encoding and readout') and the in Supplementary Information

(Section S.3). The reduction of number of virtual nodes was required by the bandwidth limitations of our modulators and AWGs, when accommodating higher information density.

Each virtual node samples a different segment of the 488-bit encoded signal over time, and their dynamics overlap asynchronously with a fractional shift defined by $\delta' \approx \theta'$, determined by the number of encoded bits and the node spacing. This behavior is stated on line 368 in the Manuscript, where the asynchronous sampling interval is defined as $\tau' = \tau / n_b + \delta'$, with τ denoting the physical round-trip time (~75 ns), n_b the number of bits per round-trip, and δ' a small desynchronization parameter. While τ remains constant, the asynchronous sampling interval τ' ensures that input data signal shifts progressively across adjacent segments and by nodes across successive roundtrips. As a result, the device generates distinct time-multiplexed transient states preserving the overall asynchronous condition necessary with p = 1 ($\delta' \approx \theta'$ in our notation) to maximize the processing capability.

We also acknowledge that some confusion may have arisen from the notation used in the earlier version of the Manuscript (line 315), where we erroneously reported $\delta = \theta/2$ with a factor of 2, which is set instead to be approximately equal to θ.

ACTION TAKEN

- We have corrected the previous notations on line 364
- We have revised the Manuscript on lines 370-373 to clarify this aspect, in which we state, '*The small desynchronization $\delta' \approx \theta'$ between the sampling and injection rates causes a progressive shift in the input seen by adjacent virtual nodes and by nodes across roundtrips, ensuring that neighboring nodes access distinct input segments over time and maintaining data correlation required for reservoir computing processing.*'

[1] https://www.nature.com/articles/s42005-021-00519-1
[2] https://opg.optica.org/oe/fulltext.cfm?uri=oe-20-20-22783&id=242389
[3] https://opg.optica.org/oe/fulltext.cfm?uri=oe-30-13-22871&id=476798
[4] https://www.nature.com/articles/s41467-024-55172-3
[5] https://opg.optica.org/optica/fulltext.cfm?uri=optica-10-11-1478&id=541462
[6] https://ieeexplore.ieee.org/abstract/document/10368205
[7] https://www.sciencedirect.com/science/article/abs/pii/S0893608012001852

We sincerely thank all reviewers once again for their constructive and thoughtful feedback. Their comments greatly helped us clarify and strengthen the Manuscript. We have carefully addressed all points raised and revised the text (in **blue color**) accordingly. We hope these changes clarify our contributions and address the points raised. We are grateful for the opportunity to strengthen the Manuscript's significance.

References

Aadhi, A. et al., 2019, Highly reconfigurable hybrid laser based on an integrated nonlinear waveguide: Optics Express, v. 27, no. 18, p. 25251, doi:10.1364/OE.27.025251.

Appeltant, L., M. C. Soriano, G. Van der Sande, J. Danckaert, S. Massar, J. Dambre, B. Schrauwen, C. R. Mirasso, and I. Fischer, 2011, Information processing using a single dynamical node as complex system: Nature communications, v. 2, p. 468, doi:10.1038/ncomms1476.

Bernstein, L., A. Sludds, C. Panuski, S. Trajtenberg-Mills, R. Hamerly, and D. Englund, 2023, Single-shot optical neural network: Science Advances, v. 9, no. 25, p. eadg7904, doi:10.1126/sciadv.adg7904.

Connelly, M. J., 2001, Wideband semiconductor optical amplifier steady-state numerical model: IEEE Journal of Quantum Electronics, v. 37, no. 3, p. 439–447, doi:10.1109/3.910455.

Dambre, J., D. Verstraeten, B. Schrauwen, and S. Massar, 2012, Information processing capacity of dynamical systems: Scientific Reports, v. 2, p. 11–20, doi:10.1038/srep00514.

Frank, S. L., and M. Čerňanský, 2008, Generalization and Systematicity in Echo State Networks: Proceedings of the Annual Meeting of the Cognitive Science Society, v. 30, no. 30.

Gu, Z., Q. Ma, X. Gao, J. W. You, and T. J. Cui, 2024, Direct electromagnetic information processing with planar diffractive neural network: Science Advances, v. 10, no. 29, p. eado3937, doi:10.1126/sciadv.ado3937.

Hülser, T., F. Köster, L. Jaurigue, and K. Lüdge, 2022, Role of delay-times in delay-based photonic reservoir computing [Invited]: Optical Materials Express, v. 12, no. 3, p. 1214–1231, doi:10.1364/OME.451016.

Kues, M., C. Reimer, B. Wetzel, P. Roztocki, B. E. Little, S. T. Chu, T. Hansson, E. A. Viktorov, D. J. Moss, and R. Morandotti, 2017, Passively mode-locked laser with an ultra-narrow spectral width: Nature Photonics, v. 11, no. 3, p. 159–162.

Liu, Y., H. Zhang, J. Liu, L. Lu, J. Du, Y. Li, Z. He, J. Chen, L. Zhou, and A. W. Poon, 2024, Parallel wavelength-division-multiplexed signal transmission and dispersion compensation enabled by soliton microcombs and microrings: Nature Communications, v. 15, no. 1, p. 3645.

Manjunath, G., and H. Jaeger, 2013, Echo State Property Linked to an Input: Exploring a Fundamental Characteristic of Recurrent Neural Networks: Neural Computation, v. 25, no. 3, p. 671–696, doi:10.1162/NECO_a_00411.

Pillai, B. S. G., B. Sedighi, K. Guan, N. P. Anthapadmanabhan, W. Shieh, K. J. Hinton, and R. S. Tucker, 2014, End-to-end energy modeling and analysis of long-haul coherent transmission systems: Journal of Lightwave Technology, v. 32, no. 18, p. 3093–3111.

Staffoli, E., G. Maddinelli, and L. Pavesi, 2024, A Silicon Photonic Neural Network for Chromatic Dispersion Compensation in 20 Gbps PAM4 Signal at 125 km and its Scalability up to 100 Gbps: Journal of Lightwave Technology.

Tang, J.-Y., B.-D. Lin, Y.-W. Shen, R.-Q. Li, J. Yu, X. He, and C. Wang, 2023, Asynchronous photonic time-delay reservoir computing: Optics Express, v. 31, no. 2, p. 2456–2466, doi:10.1364/OE.478728.

Xiong, S. et al., 2023, Investigation of stable pulse mode-locking regimes in a NALM figure-9 Er-doped fiber laser: Optics Express, v. 31, no. 1, p. 514–527, doi:10.1364/OE.476630.

REVIEWER COMMENTS

Reviewer #2 (Remarks to the Author):

The revision offers more details regarding figure-eight shaped nonlinear amplifying loop mirror architecture for photonic reservoir computing and has addressed some of my concerns. There still remain some issues that should be addressed to support this paper's claims.

1.In the revised manuscript, the authors have clarified the factors contributing to the system's scalability, such as encoding speed and the number of wavelength channels. However, it remains unclear whether the gains in computational throughput from these factors are fundamentally bounded. I recommend adding relevant analyses to help readers understand the potential upper limits of the system's capability.

We thank the reviewer for this insightful question on the fundamental throughput limits of our system.

The fiber loop latency sets the initial speed limit, since each round-trip corresponds to a single computational step. In practice, this constraint is mitigated in our approach through dense encoding and concurrent multi-channel processing, which enables many operations to occur within each loop cycle and thereby pushes the effective throughput well beyond the single-loop latency bound.

The next bottleneck to consider arises from the limited bandwidth of the encoding hardware. Specifically, our AWGs and modulators operate up to about 6.5 GHz, and we use 24 GHz modulation to generate four virtual nodes per channel. With faster encoding hardware, the dominant limitation would shift to the semiconductor optical amplifier (SOA) dynamics, which impose the fundamental bounds on device operation.

Two intrinsic SOA properties are responsible: the carrier recovery time (τ_{rec}), which sets the maximum update frequency, and the saturation energy ($\mathrm{E_{sat}}$), which fixes the maximum tolerable energy per masking cycle.

The total masking rate, defined as $f_{mask} = N_v f_{enc}$, must satisfy $f_{mask} \leq \frac{1}{\tau_{rec}}$, where here N_v is the number of virtual nodes per channel and f_{enc} is the encoding rate per channel. If this limit is exceeded, the SOA cannot recover its gain between pulses, resulting in dynamic gain compression and signal distortion. At the same time, the input energy per cycle, defined as $E_T = \frac{P_T}{f_{mask}}$, where P_T is the total circulating power must remain below E_{sat}, otherwise, nonlinear distortions such as self-phase modulation and spectral broadening appear. Additionally, exceeding this threshold would also impact the nonlinear tunability of the reservoir transfer function, which we demonstrate in this work. These two constraints act in opposite directions: raising the encoding frequency lowers the energy per symbol, which helps to satisfy the saturation condition, but it pushes closer to the recovery-time limit, while reducing the frequency relaxes the recovery requirement but increases the energy per symbol, risking saturation.

The balance between these limits determines the maximum distortion-free throughput, and combining them with the reservoir operation count leads to the expression now included in the revised Supplementary Information (Eq. S21):

$$\mathrm{S_{TOPS}^{max}} = 2 \cdot \frac{\mathrm{N_v}}{\tau_{\mathrm{rec}}} \cdot \left(\mathrm{O_n} \cdot \mathrm{N_p} \cdot \mathrm{C_f} + \mathrm{R_m} \cdot \mathrm{C'}'_{\mathrm{f}}\right),$$

where S_{TOPS}^{max} the maximum sustainable throughput, expressed in tera-operations per second (TOPS); n_c is the number of wavelength channels used for parallel processing; N_v is the number of virtual nodes per channel; τ_{rec} is the SOA carrier recovery time; O_n is the total number of reservoir operations per round-

trip (equal to 10 in our case, counting both linear and nonlinear contributions, see footnote now included in the revised Supplementary Information); N_p is the number of physical nodes in the reservoir (equal to 1 here); C_f and C'_f is a factor that accounts for arithmetic involving complex-valued variables (node states, weights, inputs). In our device, $\mathrm{S}_{\mathrm{TOPS}}^{\mathrm{max}} = 24$ TOPS per channel.

We scale by adding more parallel wavelength channels, beyond the current 5 channels. The device operates effectively with dense wavelength division multiplexing (DWDM). In standard systems, this means up to about 80 channels. We demonstrated this scalability by generating nine distinct wavelength channels through the modulation and amplification of the sidebands of a single continuous-wave (CW) laser (see Fig. S5 in the Supplementary Information). This proves that the device can support more than 5 channels simultaneously, thereby achieving higher throughput.

Importantly, our concurrent processing approach reduces latency and allows the system to operate near the fundamental limits set by SOA recovery and saturation dynamics. This represents one of the main foundations of our work.

ACTIONS TAKEN

- We moved the earlier formulation of the processing speed from the Methods section of the original Manuscript to the new Section 4.1 of the revised Supplementary Information. The discussion on speed, fundamental throughput limits, and computational scalability has been expanded, with full details (lines 211–260, Supplementary Information), to explicitly address the impact of the SOA on computational throughput. This addition clarifies the physical origin of the upper bound and its connection to both SOA dynamics and device parameters.

- We have also added a paragraph from lines 311 to 323 of the Discussion Section of the Manuscript referring to the upper bound on throughput scalability.

- To avoid ambiguity in the definition of processing speed and throughput, and following the reviewer's concern, we have added the sentence in the Manuscript from line 46 to line 50: "*Despite significant progress, RC implementations remain limited by their inherent architecture, where nodes are created and interconnected through feedback mechanisms. These mechanisms simplify the design by generating virtual nodes, but also introduce latency and restrict processing speed, defined as system throughput, i.e., the total number of operations per second across all input data streams within a single reservoir unit.*"

- We have now included a footnote below line 226 of the Supplementary Information clarifying how the definition of processing speed accounts for the nonlinear steps: *" The contribution to the nonlinear computational steps arises from the application of the nonlinear operators acting on the electric field, as defined in Eqs. (S10)–(S12). Specifically, Eq. (S10) involves two nonlinear operations, Eq. (S11) adds two more, and Eq. (S12) introduces one additional operation, for a total of five nonlinear operations. Because the electric field is complex, this number is then multiplied by a factor of 2, resulting in 10 nonlinear operations overall."*

Furthermore, while the authors state that the improvement in computational throughput requires no additional computational units, it would be valuable to discuss whether there are other hardware costs involved-for example, increased demands on lasers and AWGs, and the associated higher power consumption.

Response

We acknowledge the reviewer's point regarding peripheral hardware costs and quantify the power consumption of peripheral hardware, discussing ways to reduce it.

In the former manuscript, we had previously quantified the consumption of the input hardware, considering ~150 W for the three AWGs employed (five data channels plus one masking channel), ~0.4 W for the five IMs, and ~18 W for the CW lasers. Importantly, our current scheme employs dense encoding, where multiple bits are injected per cavity round-trip per channel, thereby increasing throughput without requiring an increase in the number of physical AWGs, IMs, or lasers. In this work, we operate with binary intensity modulation, where each symbol carries a single bit (b = 1).

In the former Manuscript, we have already reported that the computational throughput can be further increased by using additional laser sources or more advanced encoding techniques, such as quadrature-amplitude modulation (QAM). More generally, the total aggregate rate (the overall number of bits processed per second across all channels) can be expressed as

$$R_{total} = n_c \times R_{sym} \times b,$$

where R_{sym} is the symbol rate per channel, and b is the number of bits per symbol.

To increase computational throughput without impacting or even reducing the power consumption of the peripheral, one could use higher-order modulation formats such as 64-QAM (b = 6). This is sufficient to replace our current five data channels with just one at the same symbol rate. In practice, this would lower AWG usage from ~150 W to ~25 W (an ~83% reduction), reduce IM consumption from ~0.4 W to ~70 mW (also ~83% reduction), and cut the number of active laser channel from five to one (an 80% reduction), which reduces laser wall power by about 40 to 55 percent, based on vendor data and fixed overhead assumption.

With polarization-dependent multiplexing techniques, the effective modulation order would further increase, providing additional margin for throughput and reducing the need for modulation equipment even further.

Beyond modulation, another way to reduce laser overhead is to replace multiple discrete CW sources with an integrated multi-wavelength source, such as distributed-feedback laser arrays, thereby allowing a single chip to feed all channels. This eliminates per-line idle losses and, based on typical device specifications, is expected to save several watts when using one integrated source at the same optical signal-to-noise ratio and linewidth performance.

These alternatives were not implemented in the present work due to the lack of access to coherent detection equipment (e.g., high-Q photodetectors), advanced high-order AWG drivers, and integrated multi-wavelength sources in our laboratory. Nonetheless, in combination with our already employed dense encoding scheme, they represent clear future directions to mitigate peripheral overhead while leaving the reservoir core scaling unaffected.

ACTIONS TAKEN

- Together with Section S.4.1 on throughput, we moved the section on power consumption estimation (lines 301–312) to the Supplementary Information as the new Section S.4.2. Following the reviewer's recommendation, this section now provides a more detailed discussion of the encoding hardware costs and how these can be mitigated through alternative encoding strategies.

- We have also revised the text in the Discussion (lines 319–323) to highlight the role of advanced encoding techniques and dense multiplexing in both reducing hardware consumption and increasing throughput: "*For instance, with 80 channels, as typically achieved in standard DWDM, the architecture can reach a combined throughput of nearly two peta-operations per second (POPS) under binary modulation. By employing phase-sensitive processing and higher-order modulation formats, such as 64-quadrature-amplitude modulation (6 bits per symbol), the effective throughput can increase to several tens of POPS.*"

2.The revised manuscript compares the proposed system with DSP only in terms of latency and energy consumption, without detailing the performance of the two hardware platforms on the same tasks, e.g., NRMSE in the time-series prediction task and BER in the nonlinear telecom signal recovery task. Since latency and energy consumption comparisons are only meaningful under comparable task performance, adding this information would better demonstrate the advantages of the proposed method.

Response

We thank the reviewer for their comment. Since our device implements optical reservoir computing, we evaluated its performance by comparing its speed and energy consumption in detail with other optical neural network implementations, as previously reported in Table 1 of the Manuscript. In the same Table, we also report and compare classification accuracy in terms of a percentage calculated as (1- NRMSE) %, a common metric for tasks such as Mackey–Glass and $NARMA_{10}$.

On the other hand, the DSP techniques we refer to in our work are those typically used to process optical data signals and recover them from transmission distortions in telecommunication channels, with performance quantified by the BER. In the previous version of the Manuscript, we briefly compared our device's performance with DSP-based approaches for nonlinear channel recovery as a specific case study.

Recent advanced DSP methods for channel equalization, such as the deep-unfolded digital backpropagation demonstrated by Tang et al. (Optics Express 29, 36242, 2021), have achieved a BER threshold of 2×10^{-2}. Song et al. (Optics Express 30, 26774, 2022) reported a real-time FPGA-based coherent receiver using optimal interpolation clock recovery, reaching a BER of 2.4×10^{-4} at 15 Gbaud with 16-QAM. These DSP schemes rely on thousands of multipliers and operate with energy consumption in the picojoule-per-bit range. In contrast, our device achieves a BER of 2×10^{-4} while exhibiting orders of magnitude improvement in energy efficiency, operating in the few-femtojoule-per-operation range.

ACTIONS TAKEN

- We expanded and refined the discussion on DSP techniques for nonlinear channel equalization on lines 289–295. The updated text now includes quantitative examples and a detailed comparison between the performance of our approach and such DSP-based methods applied to nonlinear channel equalization: "*Advanced DSP methods, including digital backpropagation, typically report BER values near 2×10^{-2}. Real-time field-programmable gate array-based coherent receivers have achieved 2.4×10^{-4} at 15 Gbaud with 16-quadrature amplitude modulation (16-QAM)64. These*

approaches require thousands of multipliers and significantly higher power consumption, typically around 3 pJ per bit per channel, with latency between 100 ns and 1 µs, depending on the system clock. In contrast, optical signal processing methods offer a latency of 20 to 200 ps and energy consumption ranging from 0.1 to 1 pJ per bit per channel."

- We added further accuracy comparison for bit recovery tasks performed by other approaches in Table 1.

3.In the nonlinear telecom signal recovery task, the manuscript states that the proposed hardware can recover distorted signals and effectively reduce BER. However, the presented results lack a baseline comparison, making it difficult to assess whether the method's performance is truly competitive. Including relevant baselines would help readers better appreciate the method's effectiveness.

Response

We thank the reviewer for this important comment. Following our previous response to point #2 and our Action Taken, we have now reiterated some performance comparisons of our system relative to standard DSP methods used in nonlinear signal recovery (lines 289-295)

To provide further detail, we can introduce additional baseline comparisons in terms of Q-factor improvement, a performance metric used to assess the quality of a telecom (optical) signal after recovery. For instance, digital backpropagation (DBP), which numerically inverts fiber propagation, has been shown to improve the Q-factor by up to ~2 dB (Opt. Express 19, 9453–9460, 2011). This corresponds to a BER reduction from $\sim 1 \times 10^{-3}$ to the low 10^{-4} range, although DBP remains computationally expensive and scales poorly with algorithmic depth. Recent electronic neural networks have demonstrated ~1 dB Q-factor improvements (Nat. Commun. 10, 3033, 2019), reducing BER from $\sim 2\times10^{-3}$ to $\sim 1.1\times10^{-3}$, while photonic–electronic hybrid neural networks have reported ~0.8 dB gains (Nat. Electron. 4, 837–844, 2021), corresponding to a BER reduction by about a factor of 1.7.

By contrast, the recovered optical signal with our device shows a minimum BER of 2×10^{-4} after 60 km of nonlinear propagation, which corresponds to a Q-factor improvement of ~4.6 dB. This surpasses the BER and Q-factor improvements of DSP, as well as electronics-based neural network processing, while sustaining a total throughput of 20 TOPS and operating in the few-femtojoule-per-operation regime. These results demonstrate that our approach delivers comparable or superior performance, with latency and energy consumption orders of magnitude lower than those of DSP- or hybrid-based methods.

ACTIONS TAKEN

- We further expand the paragraph from line 296 to 302, including baseline comparison in terms of the Q-factor improvement of the recovered signal of our device vs some reported electronics-based and DSP techniques: "*In terms of signal quality enhancement achieved after compensation, electronic neural networks can reach a Q-factor improvement of roughly 2 dB with a BER of about 1.1×10^{-3}, while photonic–electronic hybrid networks can achieve an improvement of about 0.8 dB with a BER around 10^{-4}. Our device achieves a BER of 2×10^{-4} after 60 km of transmission at 5.4 dBm input power, corresponding to a Q-factor improvement of approximately 4.6 dB (see Fig. 5 (b)). It operates at 4.4 fJ per operation, outperforming DSP and electronic neural-network processing as well as previously reported photonic neural-network implementations, as summarized in Table 1.*"

4.In Table 1, the authors compare their work with existing photonic computing approaches, but omit prior implementations of photonic reservoirs, which are highly relevant to the proposed system. Including comparisons with such works is necessary to provide a more comprehensive evaluation.

ACTIONS TAKEN

- We have updated Table 1 to include representative examples of photonic reservoir implementations, displaying their key metrics alongside other photonic computing approaches

In conclusion, the current version still contains several issues and a second-round revision that addresses these major concerns is recommended.

We thank Reviewer #2 for their thorough assessment and constructive feedback. We appreciate the recommendation for further revisions and are confident that the updated manuscript now addresses all major concerns, strengthening the clarity and significance of our results.

Reviewer #3 (Remarks to the Author):

I appreciate the authors' thorough and thoughtful responses to our previous comments. The revised manuscript addresses all of my concerns. I recommend the acceptance of the manuscript.

We sincerely thank Reviewer #3 for their supportive evaluation and recommendation for acceptance. We are grateful for the feedback provided throughout the review process, which has helped us improve the clarity and completeness of the Manuscript

Reviewer #2 (Remarks to the Author):

I appreciate the authors’ thorough and constructive responses to the reviewers’ comments. The revised manuscript satisfactorily addresses all previous concerns, and I recommend it for publication in Nature Communications.

We thank the reviewer for their insightful comments and suggestions, which helped us improve the quality and impact of our work.
